# Whole-genome sequencing reveals an association between small genomic deletions and an increased risk of developing Parkinson’s disease

**DOI:** 10.1038/s12276-023-00952-y

**Published:** 2023-03-03

**Authors:** Ji-Hye Oh, Sungyang Jo, Kye Won Park, Eun-Jae Lee, Seung Hyun Lee, Yun Su Hwang, Ha Ra Jeon, Yeonjin Ryu, Hee Jeong Yoon, Sung-Min Chun, Chong Jai Kim, Tae Won Kim, Chang Ohk Sung, Sehyun Chae, Sun Ju Chung

**Affiliations:** 1 Department of Medical Science, Asan Medical Institute of Convergence Science and Technology, Asan Medical Center, University of Ulsan College of Medicine, Seoul, Republic of Korea; 2 Department of Pathology, Asan Medical Center, University of Ulsan College of Medicine, Seoul, Republic of Korea; 3 Department of Neurology, Asan Medical Center, University of Ulsan College of Medicine, Seoul, Republic of Korea; 4 Department of Neurology, Uijeongbu Eulji Medical Center, Eulji University School of Medicine, Uijeongbu-si, Gyeonggi-do Republic of Korea; 5 Korea Brain Bank, Korea Brain Research Institute, Daegu, Republic of Korea; 6 Department of Oncology, Asan Medical Center, University of Ulsan College of Medicine, Seoul, Republic of Korea

**Keywords:** Parkinson's disease, Genetics research

## Abstract

Single-nucleotide variants (SNVs) associated with Parkinson’s disease (PD) have been investigated mainly through genome-wide association studies. However, other genomic alterations, including copy number variations, remain less explored. In this study, we conducted whole-genome sequencing of primary (310 PD patients and 100 healthy individuals) and independent (100 PD patients and 100 healthy individuals) cohorts from the Korean population to identify high-resolution small genomic deletions, gains, and SNVs. Global small genomic deletions and gains were found to be associated with an increased and decreased risk of PD development, respectively. Thirty significant locus deletions were identified in PD, with most being associated with an increased PD risk in both cohorts. Small genomic deletions in clustered loci located in the *GPR27* region had high enhancer signals and showed the closest association with PD. *GPR27* was found to be expressed specifically in brain tissue, and *GPR27* copy number loss was associated with upregulated *SNCA* expression and downregulated dopamine neurotransmitter pathways. Clustering of small genomic deletions on chr20 in exon 1 of the *GNAS* isoform was detected. In addition, we found several PD-associated SNVs, including one in the enhancer region of the *TCF7L2* intron, which exhibited a cis-acting regulatory mode and an association with the beta-catenin signaling pathway. These findings provide a global, whole-genome view of PD and suggest that small genomic deletions in regulatory domains contribute to the risk of PD development.

## Parkinson’s disease: genetic risk factors in a Korean population

A whole-genome sequencing study of Korean individuals with Parkinson’s disease (PD) has identified several new genetic risk factors, ranging from single nucleotide variations (SNVs) to larger DNA deletions. PD is the second most prevalent neurodegenerative disease globally, but most studies have focused on SNVs in European populations. Using whole-genome sequencing, Ji-Hye Oh at the University of Ulsan, Seoul, South Korea, and co-workers were able to identify genetic differences between PD patients and healthy controls, including deletions, gains, and several new SNVs. In particular, deletions of small non-coding regions that regulate gene expression may be key contributors to PD. These results provide a whole-genome perspective on genetic risk factors for PD in a Korean population, and illuminate how a whole-genome sequencing approach may be helpful in identifying genetic factors underlying other diseases.

## Introduction

Parkinson’s disease (PD) is the second most prevalent neurodegenerative disease, affecting more than six million people worldwide^1^. PD is a multifactorial disease caused by both environmental and genetic factors. Recent studies have identified numerous genetic variants associated with the incidence and progression of PD^2^. However, most genetic studies have focused on single-nucleotide variants (SNVs) located mainly in intronic regions. To date, more than 90 independent risk-associated SNVs, including those in the *SNCA* and *LRRK2* genes, have been identified as common genetic components of PD^2–8^.

PD genetic studies have predominantly been conducted in patients of European ancestry, and other populations have largely been overlooked^2^. Foo et al. ^9^ recently conducted a large-scale genome-wide association study (GWAS) on East Asian populations and identified two novel SNVs in the *SVC2* and *WBSCR17* genes. Although SNVs provide paramount explanations of the specific phenotypes and pathogenesis of PD, high-resolution whole-genome sequencing (WGS) has revealed structural genomic variations, including copy number variations (CNVs), that affect multiple human PD phenotypes^10^. For example, CNVs are associated with familial PD but are rarely reported in sporadic PD^11^. In general, large-cohort WGS studies of CNVs may uncover additional mechanisms underlying PD pathogenesis.

Increasing evidence indicates that alterations in intergenic regions of the genome can induce diseases^6^. Indeed, many known PD risk-associated variants, including in *SNCA*, occur in noncoding regions. Soldner et al. ^12^ showed that variations in *SNCA* intronic regions have cis-acting effects that cause allele-specific changes in gene expression via deletion or exchange of disease-associated regulatory elements. This is supported by several studies highlighting the importance of noncoding variants in neurodegenerative brain diseases, most of which are located in regulatory regions, such as promoters, enhancers, and noncoding RNAs^12–14^. SNVs in noncoding regulatory regions are increasingly being associated with various diseases. Conversely, CNVs, particularly small genomic deletions or gains that affect regulatory regions, are largely uncharacterized in PD because only WGS can detect the small regions of such structural variations with high resolution.

In this study, we conducted high-resolution WGS for 310 patients with sporadic PD and 100 healthy controls to characterize PD-associated genomic variations, including CNVs. The findings were validated using WGS data from an independent secondary cohort composed of 100 patients with sporadic PD and 100 healthy controls.

## Materials and methods

### Case selection

In this prospective case‒control study, we enrolled PD patients and healthy controls at Asan Medical Center (AMC), Seoul, South Korea, between 2018 and 2020. PD diagnosis was based on the UK PD Society Brain Bank criteria^15^. Batch 1 (*n* = 210) and 2 (*n* = 100) PD cohorts were recruited from January to December in both 2018 and 2019. Healthy controls (*n* = 100) were selected from AMC visitors in 2019. Healthy controls were excluded for the following reasons: 1) a family history of PD; 2) neurological symptoms; 3) taking anticonvulsants; 4) a history of stroke or facial paralysis; or 5) diagnosed with major neurological diseases, including PD, dementia, cognitive disorders, epilepsy, or sleep apnea. The PD patients and healthy controls served as the primary cohort. For the validation (secondary) cohort, additional PD patients (*n* = 100) and healthy individuals (*n* = 100) were recruited at AMC from January to December 2020. This study was approved by the Institutional Review Board of AMC (IRB number: 2018-1510). All patients provided informed consent in accordance with the institutional review board requirements.

### DNA extraction and WGS

Genomic DNA was extracted from 610 human blood samples, and after conducting DNA quality assurance, DNA libraries were prepared using an Illumina TruSeq Nano DNA library Kit (Illumina, San Diego, CA, USA). All samples passed the quality threshold and were included for DNA library preparation. WGS was performed using an Illumina NovaSeq 6000 sequencer. Clusters were generated using paired-end 2 × 150 bp cycle sequencing reads. The binary base call files were converted to FASTQ format using Illumina bcl2fastq conversion software (version 2.20.0). The quality of the raw sequencing reads was evaluated using FastQC (version 0.11.8).

### Sequence data processing

All sequence data were processed using the Genome Analysis Toolkit (GATK) workflow (https://github.com/gatk-workflows/gatk4-data-processing)^16^. Briefly, reads were mapped to the GRCh38 reference genome via BWA-MEM (version 0.7.15) using the “-K 100,000,000 -p -v 3 -t 15 -Y” option. The MarkDuplicates and BaseRecalibrator tools (GATK, version 4.1.6) were used to mark duplicate reads and recalibrate the mapping quality, respectively. After removing duplicate reads, a mean mapped read depth of 53.6× and a mean coverage of ≥ 30× were obtained for 94% of the primary cohort genome.

### Germline variant calling and filtering

Small insertion/deletions (InDels) and SNVs were detected using the GATK workflow (https://github.com/gatk-workflows/broad-prod-wgs-germline-snps-indels)^16^. Briefly, gVCF files were generated using GATK HaplotypeCaller via the “-ERC GVCF” option. To produce the final multisample VCF, all gVCFs were pooled using the GATK GenomicsDBImport and GenotypeGVCFs (version 4.1.4) modules. Implementation of GenotypeGVCF produced multiple VCF files that were then combined into a single file containing variants from all the samples. Data in the resulting VCF file were then filtered using GATK VQSR, thereby producing high-quality variants.

To facilitate downstream variant analysis, Ensembl Variant Effect Predictor (version 101) was employed to annotate each variant by integrating several clinical and functional genome annotations, including those from dbSNP (version 153), ClinVar (version 202003), and gnomAD (version r2.1.1). In this study, WGS was used to detect all variants (SNV/SNP and InDel), which are collectively referred to as SNVs.

### SNVs associated with PD

SNVs with an allele frequency of ≥1 % were selected for conduct the GWAS. Individuals with examined clinical traits and no kinship within the selected sample set served as sample quality controls (QCs). Variant QCs were selected based on the following criteria: 1) SNVs with a minor allele frequency (MAF) of ≥0.01; 2) SNVs with a Hardy–Weinberg equilibrium (HWE) *P* > 1.0 × 10^-5^; 3) SNVs with a missing genotype rate of < 0.01; and 4) InDels not detected in segmented duplicated regions. With the assumption of the additive genetic model, GWAS was performed on QC-passed genetic markers using Scalable and Accurate Implementation of Generalized mixed model (SAIGE)^17^ software (version 0.44.6) to control for case‒control imbalances. Briefly, SAIGE comprises two steps: 1) using genotype data, the null logistic mixed model is fitted with no nongenetic covariates; and 2) single-variant association tests are performed between each genetic marker.

Variants were grouped into loci using the “-clump-p1 0.01 -clump-kb 500 -clump-r2 0.50” option of PLINK (version 1.90b)^18^. Healthy individuals recruited from AMC served as the primary control group. As an additional control, we used an external cohort composed of individuals from the Korean general population. The external cohort allele frequency data are available in Korean Genome Project (KGP), one of the largest published Korean WGS datasets^19^. We did not combine the variant calling results of the AMC healthy controls with those of the KGP dataset because the sequencing read depths and variant calling methods were different and the disease-status information of individuals in the KGP dataset was unavailable. However, combined analyses were performed via comparisons between AMC PD vs. KGP cohorts, as opposed to AMC PD vs. AMC healthy controls.

### Combining and comparing AMC PD and KGP control datasets

Data derived from PD patients (*n* = 410) were merged with the KGP dataset (*n* = 916), which consisted of unrelated individuals. VCF files containing KGP genotypes were downloaded, and multiallelic and duplicated variants were excluded. Thereafter, QCs were performed, and genetic markers were selected for each dataset using PLINK based on the following SNV filtering criteria: 1) MAF ≥ 0.01; 2) HWE *P* > 1.0 × 10^-5^; 3) missing genotype rate < 0.01; and 4) SNVs on autosomal (1–22) chromosomes. InDels were excluded from this analysis. Of the QC-passed variants, 6,612,214 intersected variants present in both datasets were used for downstream analyses. These variants were linkage disequilibrium-pruned using “-indep-pairwise 50 5 0.2” in PLINK prior to constructing principal component analysis (PCA) plots. PCA was performed using “-pca 20” in PLINK, and the first two principal components of each group or batch were visualized. GWAS was performed using SAIGE on the 6,612,214 QC-passed genetic markers with the additive genetic model assumption. During the first step, genotype data were applied to fit the null logistic mixed model with the first 10 principal components as nongenetic covariates. During the second step, each genetic marker was subjected to single-variant association tests. Finally, the variants were grouped into loci using the “-clump-kb 500 -clump-p1 1e-03 -clump-r2 0.50” option in PLINK.

### Analysis of rare genetic SNVs associated with PD

A genome-wide, gene-level sequence kernel association-optimized (SKAT-O) analysis^20^ was conducted to identify differences in the aggregate burden of rare variants between PD patients and healthy controls. We used an MAF threshold of < 0.01 and a minor allele count of ≥ 3 as filters. The analysis was performed using the R package SKAT (version 2.0.1).

### CNV analysis using different methods

Approaches that combine multiple tools have been recommended to improve germline CNV calling predictions^21,22^. Thus, we identified germline CNVs using five previously recommended^21^ tools: CNVnator^23^, GATK4-gCNV^16^, Delly2^24^, cn. MOPS^25^, and Lumpy^26^. For CNVnator, CNV calls were filtered based on the following criteria: 1) e-values (e-val1, eval2) <1.0 × 10^-3^; 2) q0 < 0.5 and q0 ≠ -1 (q0 is the fraction of reads mapped with zero quality); and 3) for deletion calls, only copy calls <0.75 of normalized read depth × (1 + q0) were used. For the other four tools, CNV calls were estimated using default parameters.

All CNV calls were combined and filtered. The five tools were used to call CNVs in each individual, and calls with > 50 % identical overlapping regions were combined using mergeSVcallers (https://github.com/zeeev/mergeSVcallers). CNVs supported by two or more tools were included in downstream analyses. These CNV regions were split at the identified CNV boundaries, and the number of copies was counted. Thereafter, CNVs overlapping ≥ 50% in centromere, telomere, or segmental duplication regions were removed^27^. Finally, associations between PD and the defined CNV regions (*n* = 120,374) for the primary cohort were evaluated using Fisher’s exact test. Only CNVnator was applied for each sample in the secondary cohort, and significantly different CNVs were identified using Fisher’s exact test. CNVs were manually curated using Integrative Genomics Viewer (IGV)^28^.

### Analysis of data downloaded from public databases

Data from 1,011 cancer cell lines of 25 tissues were downloaded from Cancer Cell Line Encyclopedia (CCLE)^1,29^. Molecular features of gene expression in cell lines with specific copy number alterations were identified. Copy number, RNA expression profiling data, and sample annotation information were downloaded from the DepMap portal (https://depmap.org/portal/download). Codependent *TCF7L2* genes were identified from CRISPR gene dependency data (DepMap 22Q2 Public + Score, Chronos) in the DepMap portal. In addition, 17,382 normalized RNA-seq gene expression (Gene TPM normalization) data from 54 normal tissue sites, including 14 brain regions, and sample annotation information were downloaded from the Genotype-Tissue Expression (GTEx, Analysis release V8) project database (https://gtexportal.org/home/datasets). The GTEx data used in this study were downloaded from the database on 09/08/2021.

To identify enhancer signals, we downloaded H3K27ac (GSM1831752) and H3K4me1 (GSM1831756) ChIP-seq data for wild-type hESC-derived neurons and H3K27ac ChIP-seq data (GSM1119249) from the substantia nigra^12,30^. These data were visualized using IGV^28^, and enhancer regions were identified based on the H3K27ac ChIP-seq results (GSM1119249)^28^. The hg19 genomic location coordinates were updated to hg38 using the UCSC LiftOver function (https://genome.ucsc.edu/cgi-bin/hgLiftOver). Hi-C-generated chromatin interactions were obtained from 3DIV, the 3D-genome interaction viewer and database^31^. HaploReg v4.1 (https://pubs.broadinstitute.org/mammals/haploreg/haploreg.php)^32^ was employed to annotate noncoding variants located in regulatory motifs, such as enhancers, across multiple tissue types.

TCF7L2 ChIP-seq data and motif analysis results for these ChIP-seq data were obtained from ENCODE (https://www.encodeproject.org). The TCF7L2 binding site target genes were predicted from the ChIP-seq data using the T-Gene algorithm^33^. Next, pathway enrichment analysis was performed for the predicted gene set using ClusterProfiler of the R package (version 3.16.1)^34^.

### Identification of molecular signatures

Enriched molecular pathways were identified via gene set enrichment analysis (GSEA)^35,36^ in subsets containing specific copy number alterations based on the C2 REACTOME gene set collection of Molecular Signatures Database (MSigDB, version 7.4)^37^. Distinctly expressed genes were selected by comparing subclasses based on copy number alterations. Gene Ontology enrichment analysis was performed using R package ClusterProfiler (version 3.16.1)^34^.

### Human leukocyte antigen typing

Human leukocyte antigen (HLA) class I and II alleles were predicted using the HLAscan algorithm^38^. HLAscan was developed to identify HLA types using the following next-generation sequencing data: whole-genome, exome, and targeted sequence data. In this study, each preprocessed BAM file was used as input, and HLAscan was executed using default parameters.

### Statistical analyses

Continuous variables were analyzed using Spearman or Pearson correlation analyses. The Wilcoxon rank-sum test was applied to evaluate significant differences between groups for continuous variables. Fisher’s exact test was utilized to evaluate significant differences between categorical variables. All two-tailed statistical analyses were performed in R version 4.0.3.

## Results

### Study design and characteristics of participants

**Fig. 1:**
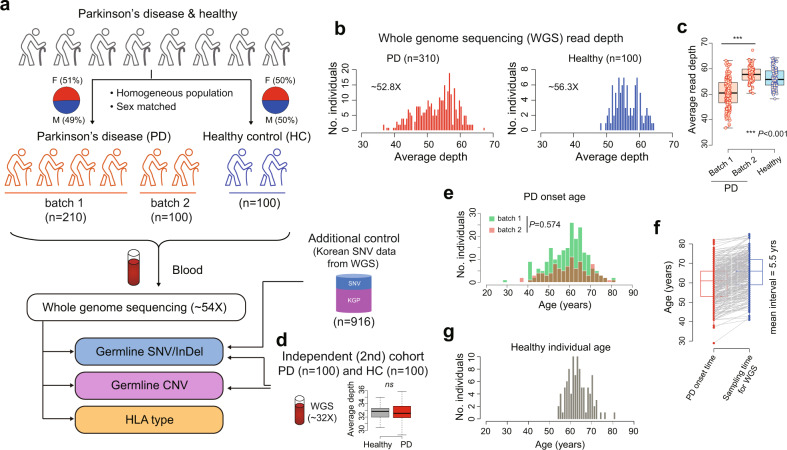
Summary of sample characteristics. **a** Overall study design and WGS. **b** Average sequencing depths of analysis-ready BAM files. **c** Relatively lower read depth of the BAM files in the batch 1 PD cohort compared to that in the batch 2 PD cohort (Wilcoxon-rank sum test). **d** WGS of an independent cohort composed of 100 PD patients and 100 healthy individuals for validation purposes (Wilcoxon-rank sum test). **e** Age of onset of PD did not differ between PD batches 1 and 2 (Wilcoxon-rank sum test). **f** Interval between blood sampling and time of onset of PD. **g** Age distribution of healthy individuals at the time of blood sampling. CNV copy number variation; HC healthy control; PD Parkinson’s disease; WGS whole-genome sequencing.

The final primary study population included 310 PD patients (*n* = 210, batch 1; *n* = 100, batch 2) and 100 healthy controls. We collected peripheral blood samples and conducted WGS, generating sequences with an average read depth of 54× (Fig. 1a). From these WGS data, we profiled germline SNVs (SNVs/InDels), CNVs and HLA molecular types. The mean depths of sequence coverage were similar between the PD (52.8×) and healthy (56.3×) individuals (Fig. 1b). However, relatively lower read depths were obtained in batch 1 than in batch 2 (Fig. 1c). This difference was taken into consideration during the identification of significant CNV differences between PD and healthy controls, as germline CNV detection sensitivity is dependent on sequencing depth^22^. In addition, the CNV results were validated using an independent cohort comprising 100 PD patients and 100 healthy individuals (Fig. 1d). Combined analysis of this secondary cohort and the primary cohort was conducted to validate suggestive SNVs.

No significant sex differences were observed between the PD patients (batch 1 and 2) and healthy controls (*P* = 0.91; Fig. 1a) or between batch 1 and 2 PD patients (*P* = 0.54; Supplementary Table 1). The mean age of onset of PD was 59.9 years (range, 29–82 years) and was similar between the patients in batches 1 and 2 (*P* = 0.57; Fig. 1e and Supplementary Table 1). The mean interval between blood sampling for WGS and PD onset was 5.5 years (Fig. 1f). The healthy individuals showed no evidence of disease following routine health examinations and brain magnetic resonance imaging (MRI). The mean blood sampling age of these individuals was 63.3 years ± 5.2 years, and their follow-up period was longer than the average age of PD onset (*P* = 0.0017; Fig. 1g).

### SNVs associated with the risk of PD

**Fig. 2:**
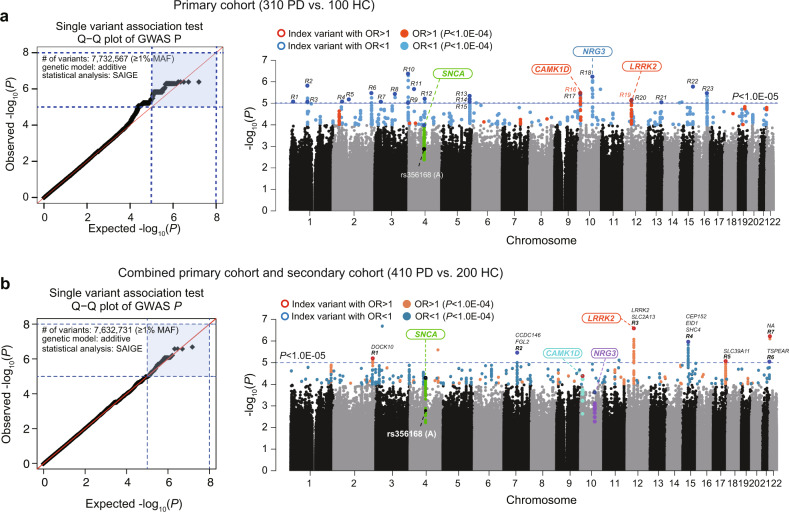
SNVs associated with risk of PD. **a**, **b** Common SNVs detected in the primary (**a**) and combined (**b**) cohorts by applying logistic mixed models to detect risk SNVs and SNVs most significantly associated with PD. HC healthy control, PD Parkinson’s disease, SNVs single-nucleotide variants.

A GWAS was conducted using the primary cohort of healthy controls recruited at AMC. On average, 3.272 × 10^6^ SNVs were detected per individual. The features of the detected germline variants are summarized in Supplementary Fig. 1a–c. No distinctive SNV batch effects were detected between batches 1 and 2 patients (Supplementary Fig. 1d). Using additive logistic mixed models, we identified 23 suggestive loci associated with PD (*P* < 1.0 × 10^-5^ based on a previous study;^39^ Fig. 2a and Supplementary Fig. 2). SNVs in the *NRG3* gene showed the strongest significant association with PD (OR = 0.23; 95 % CI = 0.13–0.41; *P* = 5.62 × 10^-7^). Additionally, *CAMK1D* and *LRRK2* SNVs were associated with PD (Fig. 2a). Although the *SNCA* loci, which are known to be associated with PD, were not among the top 23 loci identified in this study, their association with PD was significant (*P* < 0.001). When Fisher’s exact test was applied, the peak patterns were similar, and significant regions, such as *LRRK2*, *CAMK1D*, and *NRG3*, were also detected (Supplementary Fig. 3). Further analysis of GTEx-derived data showed *NRG3* and *CAMK1D* to be exclusively expressed in brain tissues (Supplementary Fig. 4a). Moreover, expression of these genes correlated significantly with *SNCA* expression in brain tissues, including the substantia nigra (Supplementary Fig. 4b). Recent studies have associated PD with *CAMK1D* and *NRG3*^40,41^, implicating these two genes as PD-associated gene candidates.

Conversely, the *LRRK2* gene was the most significant locus (Fig. 2b), as based on combined primary and secondary cohort analysis (410 PD patients and 200 healthy controls; Supplementary Fig. 5). Moreover, following combined analysis of PD (*n* = 410) and KGP healthy control (*n* = 916) datasets, known PD-associated genes, such as *SNCA* and *PRKN*, were detected as significant (*P* < 1.0 × 10^-5^; Supplementary Fig. 6). The small sample size and different testing results of these three datasets necessitate careful interpretation of the suggestive SNVs. Nevertheless, well-documented PD risk genes, including *SNCA*^7^ and *LRRK2*^42^, were also detected by WGS in Korean patients.

### Gene-level rare SNVs associated with PD

To determine whether rare genetic SNVs contribute to the existing risk of PD, we performed a genome-wide, gene-based SKAT-O analysis for rare missense and pathogenic mutations. Among 80,784 rare missense mutations, *PCDH8* exhibited the most significant association with PD (*P* = 0.00015, Supplementary Fig. 7a). Among 26,577 rare pathogenic SNVs (Supplementary Fig. 1e), the most significant association with PD was found for *SNPH* (*P* = 0.00019, Supplementary Fig. 7b). Interestingly, these two genes also displayed pronounced brain tissue-specific expression patterns (Supplementary Fig. 8a). Furthermore, analysis of GTEx data showed significant correlations between the expression levels of these genes and *SNCA* (Supplementary Fig. 8b).

### *TCF7L2* risk SNVs located in enhancer regions

**Fig. 3:**
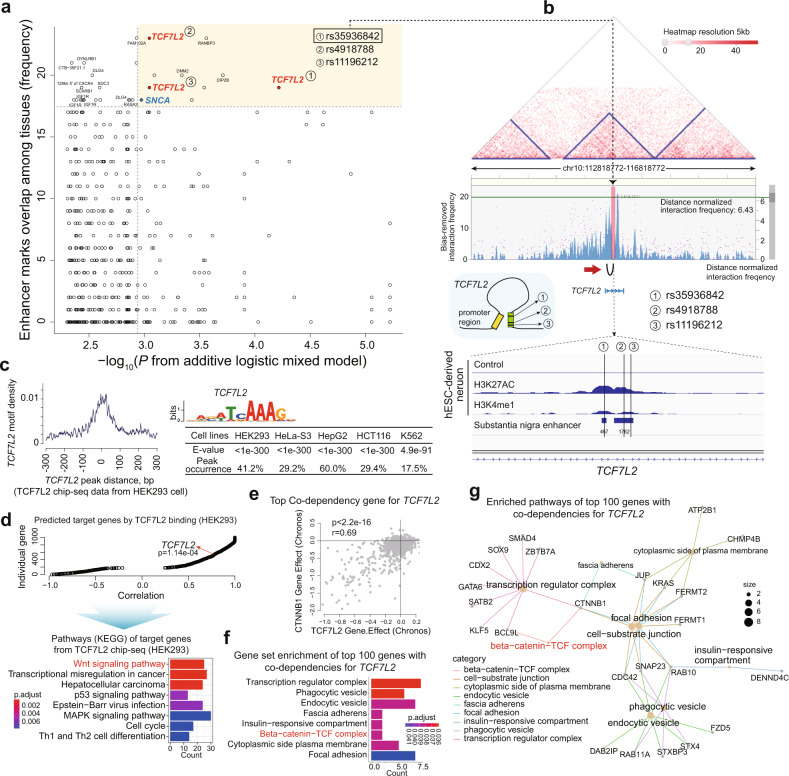
Association of SNVs in enhancer regions with risk of PD. **a** Top PD risk-associated SNVs, including those in *TCF7L2* and *SNCA*. **b** Identification and analysis of 3D genomic interactions of risk loci in *TCF7L2* gene enhancer regions. **c** Motif analysis results for TCF7L2 ChIP-seq data in multiple cell lines from ENCODE. **d** Pathway analysis using predicted TCF7L2 binding target genes from TCF7L2 ChIP-seq data in HEK292 cell lines. **e** *CTNNB1* is the top codependent gene for *TCF7L2* from DepMap data analysis (Pearson correlation analysis). **f, g** Pathway analysis using the top 100 codependent genes for *TCF7L2*. PD Parkinson’s disease; SNVs single-nucleotide variants.

SNVs in *SNCA* and *TCF7L2* enhancer regions were identified among the top genes associated with PD, with enhancer signal frequencies in multiple tissues (Fig. 3a and Supplementary Table 2). We identified enriched enhancer signals and three SNVs in *TCF7L2* using H3K27ac and H3K4m2 ChIP-seq data from hESC-derived neurons^12^ and substantia nigra tissue^30^ (Fig. 3b, lower). Analysis of brain samples from the 3D-genome interaction viewer and database^31^ showed the strongest interaction with the promoter region for the intronic loci of *TCF7L2* (Fig. 3b, red arrow). This cis-regulatory effect of *TCF7L2* is very similar to the expression regulation of *SNCA*, which leads to PD^12^. However, in contrast to brain tissue-specific expression of *SNCA*, *TCF7L2* was found to be expressed in multiple organs (Supplementary Fig. 9a). Moreover, we identified significant correlations between *SNCA* and *TCF7L2* mRNA expression in multiple brain regions, including the substantia nigra (Supplementary Fig. 9b). This finding agrees with those of previous reports showing that the transcription factor *Tcf7l2* is upregulated in Thy-aSyn mice and is significantly deregulated in PD models using neuroepithelioma cells chronically exposed to rotenone^43^.

We analyzed TCF7L2 ChIP-seq data for several cell lines from the ENCODE database (https://www.encodeproject.org). One of the best matched motifs was in *TCF7L2* (Fig. 3c), which suggests the high quality of the TCF7L2 ChIP-seq data. The predicted target genes for the TCF7L2 binding site are significantly enriched in the Wnt signaling pathways (Fig. 3d). In addition, we used DepMap data to identify *CTNNB1* as the top codependent gene of *TCF7L2* (Fig. 3e), and the beta-catenin pathway was found to be significantly associated with *TCF7L2* activity (Fig. 3f–g). The Wnt**/**beta-catenin pathway is involved in dopaminergic neuron survival and PD pathogenesis^44^. Therefore, changes in the beta-catenin pathway owing to the regulatory effect of *TCF7L2* may contribute to the development of PD.

### Small genomic deletions and increased risk of PD

**Fig. 4:**
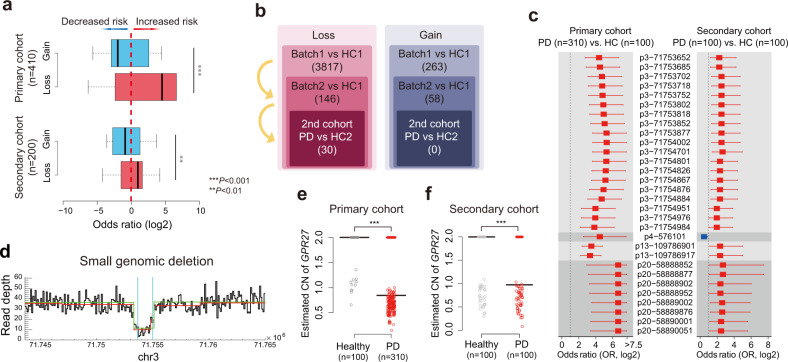
CNV and risk of PD. **a** Odds ratios of the significantly different CNVs according to CN loss or CN gain in the primary and secondary cohorts (Wilcoxon-rank sum test). **b** Selection of significant CN variations associated with the risk of PD using multiple cohorts. **c** Odds ratio of 30 significant PD risk-associated SGDs in the primary and secondary cohorts. **d** Representative image of SGDs at chromosome 3. **e**, **f** CNVnator-estimated CN of SGDs in *GPR27* of PD and healthy individuals in the primary and secondary cohorts (Wilcoxon-rank sum test). CN copy number; CNV, copy number variation; PD Parkinson’s disease; SGD small genomic deletion.

Germline copy number (CN) gains and losses across the whole genome were profiled for each individual using five different algorithms (Supplementary Fig. 10). In the primary cohort, significantly different (Fisher’s exact test *P* < 1.0 × 10^-5^) global CN gains were associated with a decreased risk of PD development (OR < 1), whereas CN losses were associated with an increased risk (OR > 1; Fig. 4a, upper). These findings were confirmed by the significantly different CNs (*P* < 0.05) in the secondary cohort; however, the sample size of the secondary cohort was smaller than that of the primary cohort (Fig. 4a, lower).

**Fig. 5:**
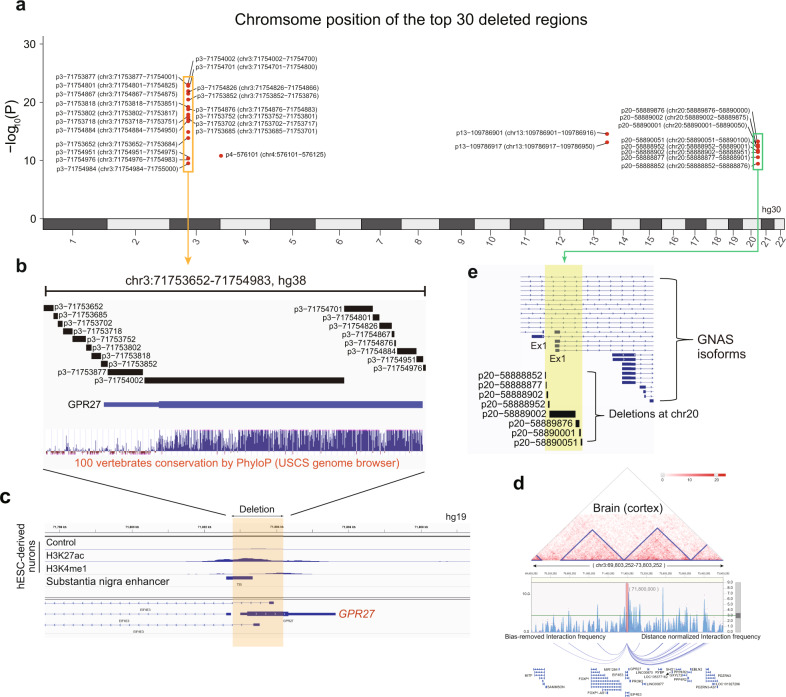
SGDs associated with risk of PD. **a** SGD loci clustered at chr3:71753652–71754983 are mainly located at *GPR27* and **b** show high conservation in multiple vertebrates. **c** High enhancer signals at the SGD loci in *GPR27*. **d** SGD loci are located in the TAD with multiple interactions. **e** SGD loci at chr20 are mainly located in the promoter region of the GNAS isoform. HC healthy control; PD Parkinson’s disease; SGD small genomic deletions; TAD topologically associated domain.

We identified the most significantly different CN loci supported by two or more programs in the primary PD cohorts. This was achieved through intersection of different CNVs among individuals in the healthy and PD batches 1 and 2, with additional filtering via the validation cohort using CNVnator (Fig. 4b). By this approach, 30 loci with small genomic deletions were detected. All these deleted loci, except one, were associated with an increased risk of PD development (OR > 1) in both the primary and secondary cohorts (Fig. 4c). Among the loci, the small genomic deletions (Fig. 4d and Supplementary Fig. 11) clustered at chr3:71753652–71,754,983 with the highest significance (*P* < 1.0 × 10^-20^) in terms of their association with the risk of PD development (OR > 1). Using the CNVnator program results, a significant difference in the small genomic deletion was observed with one copy loss in the *GPR27* region (Fig. 4e). This finding was validated using our independent secondary cohort (Fig. 4f). These small genomic deletion regions are mainly located in *GPR27* **(**Fig. 5a), a highly conserved region among 100 vertebrates (Fig. 5b). High enhancer signals in hESC-derived neurons and substantia nigra tissue were found for this region (Fig. 5c). Using the 3D genome looping structure, the region was shown to be located in close proximity to the topologically associating domain (TAD), demonstrating multiple interactions with other genomic regions in brain tissue (Fig. 5d and Supplementary Fig. 12). Disruptions in the TAD have been implicated in the pathogenesis of rare diseases^45^. Interestingly, clustered small genomic deletions at chr20:58888852–58890100 are located in exon 1 of the *GNAS* isoform (Fig. 5e), which suggests that a specific isoform of *GNAS* contributes greatly to brain diseases.

### Pathogenesis of *GPR27* CN loss increasing the risk of PD development

**Fig. 6:**
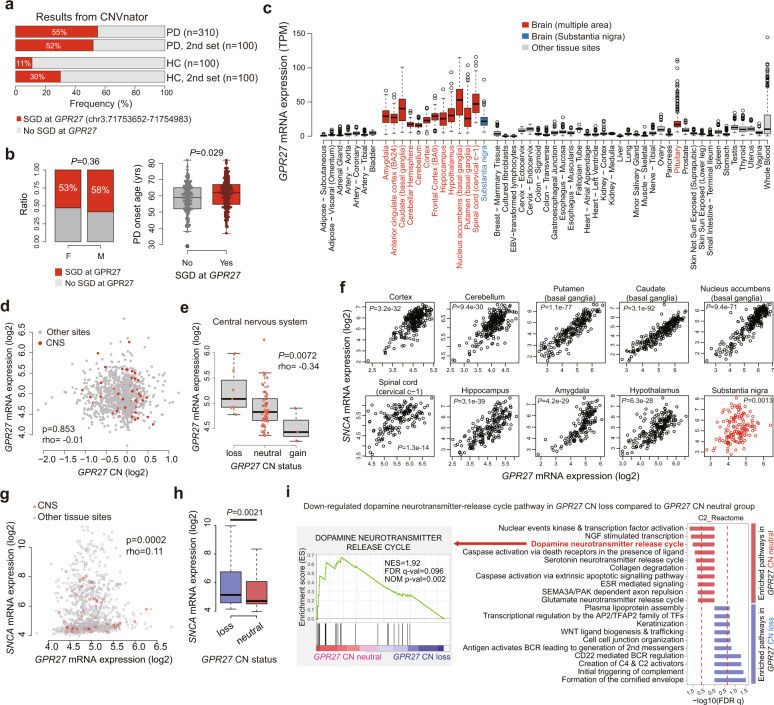
Pathogenesis of *GPR27* CN loss in PD. **a** Frequencies of SGDs in *GPR27* between PD patients and healthy controls. **b** Associations between patient sex and age of onset of PD based on SGDs in the primary cohort (Fisher’s exact test and Wilcoxon-rank sum test, respectively). **c** *GPR27* showed significant brain tissue-specific expression patterns, including those in the substantia nigra (data obtained from the GTEx project). **d** No correlation between *GPR27* CN loss and *GPR27* mRNA expression. Analysis (Spearman correlation test) based on data from CCLE. **e** Negative correlation between *GPR27* mRNA expression and *GPR27* CN status in the CNS. Analysis (Spearman correlation test) based on data from CCLE. **f**, **g** Significant correlations between *SNCA* and *GPR27* mRNA expression in brain tissues, including those in the substantia nigra (GTEx database) (**f**) and in cancer cell lines (CCLE data) (**g**) (Spearman correlation test). **h** Higher *SNCA* mRNA expression associated with *GPR27* CN loss. Analysis (Wilcoxon-rank sum test) based on data from CCLE. **i** Differences in the dopaminergic pathway between *GPR27* CN loss and *GPR27* CN neutral samples, indicating a downregulated dopaminergic pathway in the *GPR27* CN loss group. Gene set enrichment analysis was based on CCLE data. CCLE Cancer Cell Line Encyclopedia, CN copy number, CNS central nervous system, SGD small genomic deletion, PD Parkinson’s disease.

The overall frequency of the small deletion estimated using CNVnator was 50% in PD patients and 11–30% in healthy individuals (Fig. 6a). We further confirmed the frequencies of CN deletions, including those in *GPR27*, using Database of Genomic Variants (Supplementary Fig. 13). Although the deletion was not associated with sex, it was associated with age, as individuals with this small genomic deletion developed PD at an older age (*P* = 0.029, Fig. 6b). Analysis of GTEx data showed brain tissue-specific expression patterns of *GPR27* (Fig. 6c), suggesting that germline CNVs in this gene mainly affect the brain.

To determine the functional relationship between *GPR27* CN loss and PD, we analyzed gene expression and CN data obtained from CCLE (cancer cell lines) as well as gene expression data obtained from the GTEx database (normal tissues). Furthermore, we investigated the effect of CNVs on expression levels of these genes. Analysis of CCLE data showed that CN alterations in *GPR27* had no effect on gene expression levels (Fig. 6d). However, analysis of CCLE data showed a significant negative correlation between CNVs and gene expression in the central nervous system (CNS) (Fig. 6e). According to GTEx data, *GPR27* expression correlated significantly with *SNCA* expression in various normal brain tissues, including the substantia nigra (Fig. 6f), and this correlation was also demonstrated using CCLE data (Fig. 6g). Consequently, higher *SNCA* expression was noted in the cell line group with *GPR27* CN loss (Fig. 6h), suggesting that *SNCA* mediates the signaling pathway through which *GPR27* CN loss contributes to PD pathogenesis. To obtain additional evidence supporting the pathological role of *GPR27* CN loss, we conducted pathway analyses using data obtained from the CCLE database. GSEA using CCLE data from CNS samples revealed a downregulated dopamine neurotransmitter release cycle in the *GPR27* CN loss group compared to that in the *GPR27* CN neutral group (Fig. 6i). Collectively, these findings suggest that germline *GPR27* CN loss contributes to PD development.

### HLA type and PD

High-resolution HLA molecular typing was performed using WGS data for PD patients and healthy individuals. The overall frequency of the HLA molecular types identified in this study concurred with previously reported results from an analysis of 5,802 Koreans (Supplementary Fig. 14a)^46^. Various HLA molecular types were present in patients with PD; however, we found no statistically significant differences in their frequencies between PD patients and healthy individuals (FDR q > 0.05) (Supplementary Fig. 14b).

## Discussion

Many studies have explored the causal factors of PD and identified multiple PD susceptibility loci, providing evidence the genetic origin of this disease^2–8^. Many of these studies were GWAS based and focused on SNVs. Although SNVs are most frequently associated with disease pathogenesis, other genetic variations, such as CNVs, are also contributing factors, yet studies on these structural variations in PD are limited. WGS allows for high-resolution detection of CNVs and small CNV regions. Using high-read-depth WGS data, we for the first time identified that global small genomic deletions are associated with an increased risk of PD development. In contrast, global small genomic gains were found to be associated with a decreased risk of PD development. We hypothesized that these different contributions to PD risk are related to advantages and disadvantages in the evolutionary development of the human brain. Among the deletions, we detected clustered small genomic deletions located in the *GPR27* region of PD patients. GPR27 is a G protein-coupled receptor involved in neuronal plasticity and energy metabolism and is expressed in the brain^47^. In particular, the small deleted regions are enhancers located in the TAD, suggesting that their alterations may affect protein production, unlike other SNVs in noncoding regions.

We also confirmed *GPR27* CN loss to be associated with upregulated *SNCA* expression and a downregulated dopamine neurotransmitter release cycle. Transcriptional or posttranscriptional upregulation of SNCA has been suggested to cause PD^48^. Although the causal relationship between *GPR27* CN loss and upregulated SNCA expression is unclear, we speculate that the *GPR27* loss precedes alteration of SNCA expression, considering their role in signal transduction. Reduced dopamine neurotransmitter levels in PD contribute to the manifestation of motor and nonmotor symptoms of PD, though it had been suggested as the consequence of alpha-synuclein deposition and dopaminergic neuronal loss. Whether GPR27 CN loss contributes to symptom development in addition to neuronal death needs further investigation.

Furthermore, several PD-associated candidate SNVs were identified, specifically intronic SNVs located within *TCF7L2* enhancer regions. A cis-regulatory expression mode similar to the *SNCA* regulatory mechanism in PD has been suggested for these SNVs^12^. We observed a significant correlation between *TCF7L2* and *SNCA* in brain tissues. In addition, *TCF7L2* has distinct functions in the development and maintenance of thalamic and midbrain neurons^49^. The *TCF7L2* ChIP-seq and gene dependency data suggest the potential role of the beta-catenin pathway in PD pathogenesis. The Wnt/beta-catenin pathway regulates cell proliferation and differentiation, apoptosis, and inflammation and has been investigated in cancer^50^. Recent studies have suggested the association between the Wnt pathway and mitochondrial dynamics, the cell cycle, and inflammatory and oxidative pathways, and the connection between the Wnt pathway and the pathogenesis of PD has been highlighted^51^. Our results suggest that *TCF7L2* SNVs contribute to the development of PD through Wnt/β-catenin signaling. In addition to *TCF7L2*, SNVs in other genes, including *NRG3*, *CAMK1D*, *PCDH8*, and *SNPH*, were observed to be associated with PD development. These genes exhibited a specific expression pattern in the brain and were associated with *SNCA* expression. These findings suggest that *SNCA* plays a major role in PD development.

Our study has several limitations. First, the number of included samples was relatively limited to detect PD risk-associated SNVs. Nevertheless, candidate regions, specifically CNV regions, can be attributed to PD pathogenesis, though additional validation studies are needed. Second, the sequencing depths of batches 1 and 2, healthy controls, and the validation cohort differed. Detection of CNVs may be influenced by the sequencing quality (e.g., sequencing depth, coverage, sample quality, etc.); therefore, we applied strict filtering criteria (i.e., comparison case and batch control). In addition, batch effects might arise from various external factors, such as drug treatments, sample collection and storage time, and detection algorithms^22,52,53^. Several medications reportedly cause CNV via genotoxicity^52,53^. However, to the best of our knowledge, no such reports currently exist for PD medications. The sample collection and storage time (sample storage period) are important factors in the batch effect and affect the quality of sequencing data, thus impacting CNV detection. Therefore, we aimed to minimize this confounding effect by dividing the cohort (sample batch) according to the sample collection time. Accordingly, we mainly analyzed the primary cohort, for which there was a higher sequencing depth and larger sample size than for the secondary cohort. Nevertheless, the secondary cohort was used for validation, and combined analysis of both cohorts produced more robust results for SNVs in genes, such as *LRRK2*. Moreover, use of the large KGP dataset as a healthy control group facilitated the additional identification of known PD genes, such as *SNCA* and *PRKN* (Parkin). For SNV analysis, a *P* value cutoff of 1.0 × 10^-8^ may have provided more significant results than the relatively low cutoff of 1.0 × 10^-5^ used in this study. Careful interpretation of the suggestive SNVs identified in this study is recommended.

For the primary cohort, WGS targeted an average mappable depth of 40×. Although the read depth was similar for the PD and healthy control cohorts, there was a slight difference between them due to the relatively low mean depth of the batch 1 PD cohort (Fig. 1b–c). The samples of the batch 1 cohort were collected earlier than those of the batch 2 cohorts, which may have affected the sequencing depth due to long-term sample storage and resulting DNA degradation.

Regarding the WGS data, CNV calling was affected by the detection programs, though the CNV detection accuracy can be improved by combining various callers^22,54^. Therefore, we combined five types of callers to detect CNVs. The accuracy of the detected CNVs in this study was considered to be high after evaluating batch effects, such as sequencing depth and sample collection time. PD risk-associated CNVs were more notable in the primary cohort (Fig. 4a). This may be ascribed to the higher sequencing depth and statistical power owing to the larger sample size (*n* = 410) compared to the secondary cohort (*n* = 200). Moreover, only one CNV caller was used for the secondary cohort, which may also have contributed to these differences; however, reproduced results using these different methods may increase the robustness of the CNV results.

In conclusion, we analyzed the association between PD and germline CNVs, specifically in regulatory domains, to gain a better understanding of PD pathogenesis. This is the first WGS study of PD conducted in a large patient cohort. We identified the role of CNV patterns in PD pathogenesis and associations between small genomic deletions and PD development. The findings of this study suggest that high-resolution WGS analyses of structural genomic variations are promising for identifying the currently unknown causes of PD.

## Supplementary information

Supplementary information

## Data Availability

The raw sequencing data supporting the study findings have not been deposited in a public repository to safeguard participant privacy but are available from the corresponding author upon reasonable request. All SNVs with summary statistics identified in the combined analysis (Fig. 2b) have been deposited in the European Genome-Phenome Archive (EGA: https://ega-archive.org/) with Dataset ID (EGAD00001009775).

## References

[1] The Cancer Cell Line Encyclopedia Consortium & The Genomics of Drug Sensitivity in Cancer Consortium. Pharmacogenomic agreement between two cancer cell line data sets. *Nature* **528**, 84–87 (2015). 10.1038/nature15736 PMC6343827 26570998

[2] Blauwendraat C, Nalls MA, Singleton AB (2020). The genetic architecture of Parkinson’s disease. Lancet Neurol..

[3] Chartier-Harlin MC (2004). Alpha-synuclein locus duplication as a cause of familial Parkinson’s disease. Lancet.

[4] Ibáñez P (2004). Causal relation between alpha-synuclein gene duplication and familial Parkinson’s disease. Lancet.

[5] Zhang PL, Chen Y, Zhang CH, Wang YX, Fernandez-Funez P (2018). Genetics of Parkinson’s disease and related disorders. J. Med. Genet..

[6] Benhelli-Mokrani H (2018). Genome-wide identification of genic and intergenic neuronal DNA regions bound by Tau protein under physiological and stress conditions. Nucleic Acids Res.

[7] Nalls MA (2014). Large-scale meta-analysis of genome-wide association data identifies six new risk loci for Parkinson’s disease. Nat. Genet..

[8] Simón-Sánchez J (2009). Genome-wide association study reveals genetic risk underlying Parkinson’s disease. Nat. Genet..

[9] Foo JN (2020). Identification of Risk Loci for Parkinson Disease in Asians and Comparison of Risk Between Asians and Europeans: A Genome-Wide Association Study. JAMA Neurol..

[10] Iafrate AJ (2004). Detection of large-scale variation in the human genome. Nat. Genet..

[11] Toft M, Ross OA (2010). Copy number variation in Parkinson’s disease. Genome Med.

[12] Soldner F (2016). Parkinson-associated risk variant in distal enhancer of α-synuclein modulates target gene expression. Nature.

[13] Frydas A, Wauters E, van der Zee J (2021). & Van Broeckhoven, C. Uncovering the impact of noncoding variants in neurodegenerative brain diseases. Trends Genet..

[14] Kikuchi M (2019). Enhancer variants associated with Alzheimer’s disease affect gene expression via chromatin looping. BMC Med. Genom..

[15] Hughes AJ, Daniel SE, Kilford L, Lees AJ (1992). Accuracy of clinical diagnosis of idiopathic Parkinson’s disease: a clinico-pathological study of 100 cases. J. Neurol. Neurosurg. Psychiatry.

[16] McKenna A (2010). The Genome Analysis Toolkit: a MapReduce framework for analyzing next-generation DNA sequencing data. Genome Res.

[17] Zhou W (2018). Efficiently controlling for case-control imbalance and sample relatedness in large-scale genetic association studies. Nat. Genet..

[18] Purcell S (2007). PLINK: a tool set for whole-genome association and population-based linkage analyses. Am. J. Hum. Genet..

[19] Jeon S (2020). Korean Genome Project: 1094 Korean personal genomes with clinical information. Sci. Adv..

[20] Lee S (2012). Optimal unified approach for rare-variant association testing with application to small-sample case-control whole-exome sequencing studies. Am. J. Hum. Genet..

[21] Gabrielaite M (2021). A comparison of tools for copy-number variation detection in germline whole exome and whole genome sequencing data. Cancers (Basel).

[22] Trost B (2018). A Comprehensive Workflow for Read Depth-Based Identification of Copy-Number Variation from Whole-Genome Sequence Data. Am. J. Hum. Genet..

[23] Abyzov A, Urban AE, Snyder M, Gerstein M (2011). CNVnator: an approach to discover, genotype, and characterize typical and atypical CNVs from family and population genome sequencing. Genome Res.

[24] Rausch T (2012). DELLY: structural variant discovery by integrated paired-end and split-read analysis. Bioinformatics.

[25] Klambauer G (2012). cn.MOPS: mixture of Poissons for discovering copy number variations in next-generation sequencing data with a low false discovery rate. Nucleic Acids Res.

[26] Layer RM, Chiang C, Quinlan AR, Hall IM (2014). LUMPY: a probabilistic framework for structural variant discovery. Genome Biol..

[27] Yilmaz F (2021). Genome-wide copy number variations in a large cohort of bantu African children. BMC Med. Genom..

[28] Robinson JT (2011). Integrative genomics viewer. Nat. Biotechnol..

[29] Barretina J (2012). The Cancer Cell Line Encyclopedia enables predictive modelling of anticancer drug sensitivity. Nature.

[30] Vermunt MW (2014). Large-scale identification of coregulated enhancer networks in the adult human brain. Cell Rep..

[31] Kim K (2021). 3DIV update for 2021: a comprehensive resource of 3D genome and 3D cancer genome. Nucleic Acids Res..

[32] Ward LD, Kellis M (2012). HaploReg: a resource for exploring chromatin states, conservation, and regulatory motif alterations within sets of genetically linked variants. Nucleic Acids Res.

[33] O’Connor T, Grant CE, Bodén M, Bailey TL (2020). T-Gene: improved target gene prediction. Bioinformatics.

[34] Yu G, Wang LG, Han Y, He Q (2012). Y. clusterProfiler: an R package for comparing biological themes among gene clusters. OMICS.

[35] Mootha VK (2003). PGC-1alpha-responsive genes involved in oxidative phosphorylation are coordinately downregulated in human diabetes. Nat. Genet..

[36] Subramanian A (2005). Gene set enrichment analysis: a knowledge-based approach for interpreting genome-wide expression profiles. Proc. Natl Acad. Sci. USA..

[37] Vellios N, van der Zee K (2020). Dataset on cigarette smokers in six South African townships. Data Brief..

[38] Ka S (2017). HLAscan: genotyping of the HLA region using next-generation sequencing data. BMC Bioinforma..

[39] Wang X (2012). Genome-wide association scan of dental caries in the permanent dentition. BMC Oral. Health.

[40] Ou GY, Lin WW, Zhao WJ (2021). Neuregulins in Neurodegenerative Diseases. Front. Aging Neurosci..

[41] Korecka JA (2019). Neurite Collapse and Altered ER Ca(2+) Control in Human Parkinson Disease Patient iPSC-Derived Neurons with LRRK2 G2019S Mutation. Stem Cell Rep..

[42] Li JQ, Tan L, Yu JT (2014). The role of the LRRK2 gene in Parkinsonism. Mol. Neurodegener..

[43] Cabeza-Arvelaiz Y (2011). Analysis of striatal transcriptome in mice overexpressing human wild-type alpha-synuclein supports synaptic dysfunction and suggests mechanisms of neuroprotection for striatal neurons. Mol. Neurodegener..

[44] Marchetti B (2018). Wnt/β-Catenin Signaling Pathway Governs a Full Program for Dopaminergic Neuron Survival, Neurorescue and Regeneration in the MPTP Mouse Model of Parkinson’s Disease. Int. J. Mol. Sci..

[45] McArthur E, Capra JA (2021). Topologically associating domain boundaries that are stable across diverse cell types are evolutionarily constrained and enriched for heritability. Am. J. Hum. Genet..

[46] Park HJ (2016). HLA Allele Frequencies in 5802 Koreans: Varied Allele Types Associated with SJS/TEN According to Culprit Drugs. Yonsei Med. J..

[47] Pillaiyar T (2021). Structure-activity relationships of agonists for the orphan G protein-coupled receptor GPR27. Eur. J. Med. Chem..

[48] Tagliafierro L, Chiba-Falek O (2016). Up-regulation of SNCA gene expression: implications to synucleinopathies. Neurogenetics.

[49] Lee S, Lee CE, Elias CF, Elmquist JK (2009). Expression of the diabetes-associated gene TCF7L2 in adult mouse brain. J. Comp. Neurol..

[50] Silva-García O, Valdez-Alarcón JJ, Baizabal-Aguirre VM (2019). Wnt/β-Catenin Signaling as a Molecular Target by Pathogenic Bacteria. Front. Immunol..

[51] Marchetti B (2020). Parkinson’s disease, aging and adult neurogenesis: Wnt/β-catenin signalling as the key to unlock the mystery of endogenous brain repair. Aging Cell.

[52] Todd RT, Selmecki A (2020). Expandable and reversible copy number amplification drives rapid adaptation to antifungal drugs. Elife.

[53] Arlt MF (2018). Effects of hydroxyurea on CNV induction in the mouse germline. Environ. Mol. Mutagen..

[54] Coutelier M (2022). Combining callers improves the detection of copy number variants from whole-genome sequencing. Eur. J. Hum. Genet..

