# Supplementary material for: Whole-genome sequencing reveals an association between small genomic deletions and an increased risk of developing Parkinson’s disease
Source: Exp Mol Med. 2023 Mar 3;55(3):555–64. doi: 10.1038/s12276-023-00952-y (PMC10073127; doi:10.1038/s12276-023-00952-y)
Supplement: Supplementary file 1 — Supplementary information [file 12276_2023_952_MOESM1_ESM.pdf]

Supplementary information

Whole genome sequencing reveals an association between small genomic deletions and an increased risk of developing Parkinson's disease

Ji-Hye Oh, Sungyang Jo, Kye Won Park, Eun-Jae Lee, Seung Hyun Lee, Yun Su Hwang, Ha Ra Jeon, Yeonjin Ryu, Hee Jeong Yoon, Sung-Min Chun, Chong Jai Kim, Tae Won Kim, Chang Ohk Sung, Sehyun Chae, Sun Ju Chung

Contents

1. Supplementary Figures -- 2

2. Supplementary Tables -- 16

Supplementary Fig. 1

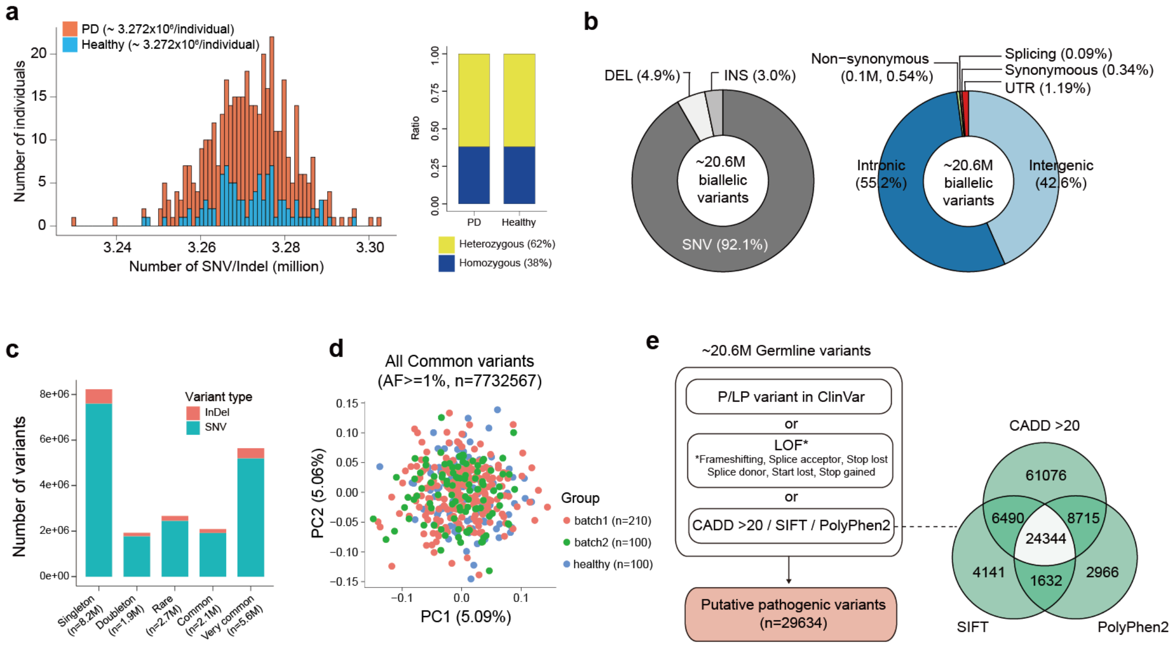

Supplementary Fig. 1. Summary of variant calling from WGS data of primary cohort (310 PD patients and 100 healthy individuals). **(a)** Frequencies of all detected variants. **(b)** Frequencies of different variant types and their genomic locations. **(c)** Number of variants according to allele frequencies. **(d)** PCA plot of variants detected in PD and healthy cohorts. (e) Detection of pathogenic variants. LOF, loss of function; PD, Parkinson's disease; WGS, whole genome sequencing.

Supplementary Fig. 2

No	CHR	RANGE	KB	N	GENES IN RANGE	Most significant variant in same linkage disequilibrium block							
						Variant identifier	Hugo symbol	Consequence	dbSNP	OR (95% CI)	*P*	A1/A2	MAF
R1	1	chr1:17370887..17469684	98.8	2	*PADI6,RCC2*	chr1_17370887_A_T	*PADI6*	intergenic	rs540362756	0.04 (0.01-0.16)	8.2E-06	T/A	0.015
R2	1	chr1:99953732..100298312	345	321	*DBT,HIAT1,LOC100506007, LRRC39,MIR553,RTCA,SASS6, SLC35A3,TRMT13*	chr1_100073894_A_T	*MFSD14A*	intronic	rs17121829	0.03 (0.01-0.11)	1.5E-06	T/A	0.013
R3	1	chr1:100190027..100298391	108	24	*DBT,LOC100506007,MIR553,RTCA*	chr1_100244533_T_G	*DBT*	intronic	rs3131839	0.06 (0.02-0.21)	8.3E-06	T/G	0.02
R4	2	chr2:52988297..52997188	8.89	7		chr2_52997188_T_C	*Unknown*	intergenic	rs9973718	0.04 (0.01-0.16)	8.2E-06	C/T	0.015
R5	2	chr2:91931237..91931240	0	2		chr2_91931237_T_C	*SLC9B1P2*	intronic	rs1378953624	0.23 (0.12-0.44)	6.4E-06	C/T	0.08
R6	2	chr2:222955279..223035596	80.3	69		chr2_223005102_G_A	*Unknown*	intergenic	rs11681653	0.17 (0.08-0.36)	3.3E-06	G/A	0.057
R7	3	chr3:35266730..35421094	154	3	*LOC101928135*	chr3_35421094_G_T	*Unknown*	intergenic	rs189905377	0.06 (0.02-0.21)	8.3E-06	T/G	0.02
R8	3	chr3:117362187..117390183	28	3		chr3_117364574_A_C	*Unknown*	intergenic	rs138625451	0.15 (0.07-0.33)	3.6E-06	C/A	0.048
R9	3	chr3:193815914..193818241	2.33	7		chr3_193817843_G_GT	*ENSR00001077711*	intergenic	rs138146003	0.4 (0.26-0.6)	9.3E-06	GT/G	0.2
R10	3	chr3:193818464..193839512	21	33		chr3_193821331_T_A	*ENSR00000713174*	intergenic	rs1165639	0.4 (0.28-0.57)	4.1E-07	A/T	0.295
R11	4	chr4:30661782..30982620	321	4	*PCDH7*	chr4_30661782_G_C	*Unknown*	intergenic	rs181470088	0.04 (0.01-0.14)	2.1E-06	C/G	0.016
R12	4	chr4:92157908..92559399	401	8	*GRID2*	chr4_92474818_A_G	*GRID2*	intronic	rs150384524	0.03 (0.01-0.13)	6E-06	G/A	0.012
R13	5	chr5:163618938..163647644	28.7	47		chr5_163636957_A_G	*Unknown*	intergenic	rs17062104	0.37 (0.24-0.56)	4.4E-06	G/A	0.18
R14	5	chr5:163638722..163692066	53.3	4		chr5_163638722_C_T	*Unknown*	intergenic	rs186783666	0.16 (0.08-0.36)	6.3E-06	T/C	0.048
R15	5	chr5:163650687..163697614	46.9	22		chr5_163650687_C_T	*Unknown*	intergenic	rs113014072	0.28 (0.16-0.49)	8.4E-06	T/C	0.096
R16	10	chr10:12464347..12475846	11.5	15	*CAMK1D*	chr10_12469763_A_G	*CAMK1D*	intronic	rs2768412	2.23 (1.59-3.12)	3.2E-06	G/A	0.312
R17	10	chr10:12467132..12477576	10.4	7	*CAMK1D*	chr10_12477576_C_T	*CAMK1D*	intronic	rs7920524	0.47 (0.34-0.65)	3.6E-06	T/C	0.487
R18	10	chr10:82746709..82861278	115	25	*NRG3*	chr10_82818088_G_A	*NRG3*	intronic	rs118127986	0.23 (0.13-0.41)	5.6E-07	A/G	0.091
R19	12	chr12:40107231..40304884	198	72	*LRRK2*	chr12_40147356_C_A	*LINC02555*	intergenic	rs2638270	2.11 (1.52-2.93)	7.3E-06	C/A	0.38
R20	12	chr12:42481266..42527289	46	17	*PRICKLE1*	chr12_42515321_C_A	*PRICKLE1*	intronic	rs149395950	0.35 (0.22-0.55)	6.7E-06	A/C	0.138
R21	13	chr13:97886598..98010500	124	2	*IPO5*	chr13_98010500_T_C	*IPO5*	intronic	rs140280266	0.05 (0.01-0.19)	8.8E-06	C/T	0.017
R22	15	chr15:92953240..93012601	59.4	3	*CHD2*	chr15_92953240_G_A	*CHD2*	intronic	rs138455747	0.08 (0.03-0.22)	1.6E-06	A/G	0.024
R23	16	chr16:71997961..72203636	206	235	*DHODH,DHX38,HP,HPR, PKD1L3,PMFBP1,TXNL4B*	chr16_72045090_TG_T	*TXNL4B*	intronic	rs35283911	0.43 (0.3-0.61)	3.3E-06	T/TG	0.292

Supplementary Fig. 2. Top 23 loci associated with Parkinson's disease identified in the primary cohort using logistic mixed models.

Supplementary Fig. 3

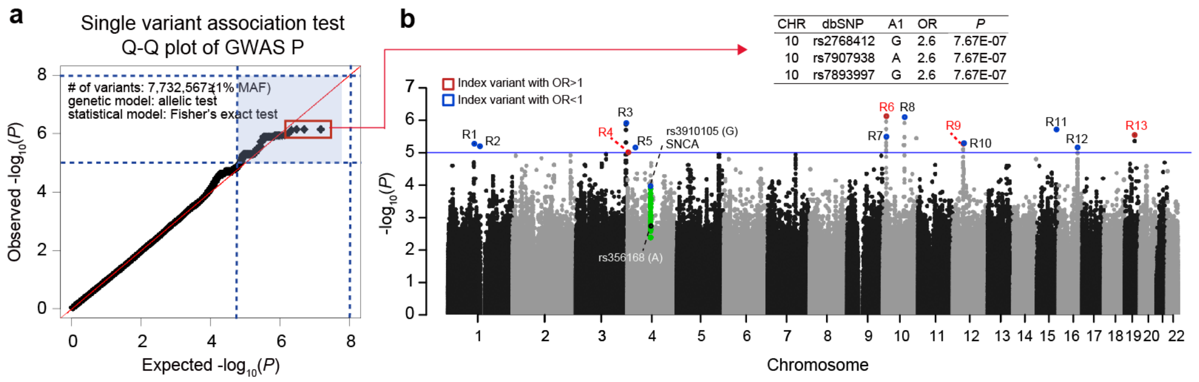

c

NO	CHR	RANGE	KB	N	GENES IN RANGE	Most significant variant in same linkage disequilibrium block									
						Variant identifier	Hugo symbol	Consequence	dbSNP	A1/A2	MAF	OR, SAIGE (additive)	P, SAIGE (additive)	OR, fisher exact (allelic)	P, fisher exact (allelic)
R1	1	chr1:99953732-100298312	345	321	*DBT,HIAT1,LOC100506007, MIR553,RTCA,SASS6, SLC35A3,TRMT13*	chr1_100073894_A_T	*MFSD14A*	intronic	rs17121829	T/A	0.013	0.03	1.48E-06	0.03	5.42E-06
R2	1	chr1:121459491-121737055	278	2	*EMBP1*	chr1_121737055_G_T	*Unknown*	intergenic	rs113464816	T/G	0.046	0.18	1.04E-05	0.22	6.48E-06
R3	3	chr3:193818464-193839512	21	33		chr3_193821331_T_A	*ENSR00000713174*	intergenic	rs1165639	A/T	0.295	0.40	4.12E-07	0.43	1.25E-06
R4	4	chr4:3493556-3534385	40.8	8	*DOK7,LRPAP1*	chr4_3534385_T_C	*LRPAP1*	intergenic	rs2073502	C/T	0.139	2.45	8.54E-05	3.83	9.99E-06
R5	4	chr4:30661782-30982620	321	4	*PCDH7*	chr4_30661782_G_C	*Unknown*	intergenic	rs181470088	C/G	0.016	0.04	2.12E-06	0.06	7.08E-06
R6	10	chr10:12464347-12475846	11.5	15	*CAMK1D*	chr10_12469763_A_G	*CAMK1D*	intronic	rs2768412	G/A	0.312	2.23	3.22E-06	2.61	7.67E-07
R7	10	chr10:12467132-12477576	10.4	7	*CAMK1D*	chr10_12477576_C_T	*CAMK1D*	intronic	rs7920524	T/C	0.487	0.47	3.57E-06	0.46	3.27E-06
R8	10	chr10:82746709-82861278	115	25	*NRG3*	chr10_82818088_G_A	*NRG3*	intronic	rs118127986	A/G	0.091	0.23	5.62E-07	0.29	8.20E-07
R9	12	chr12:40107231-40304884	198	72	*LRRK2*	chr12_40147356_C_A	*LINC02555*	intergenic	rs2638270	C/A	0.381	2.11	7.29E-06	2.27	5.43E-06
R10	12	chr12:42481266-42527289	46	17	*PRICKLE1*	chr12_42515321_C_A	*PRICKLE1*	intronic	rs149395950	A/C	0.138	0.35	6.73E-06	0.37	5.20E-06
R11	15	chr15:92953240-93012601	59.4	3	*CHD2*	chr15_92953240_G_A	*CHD2*	intronic	rs138455747	A/G	0.024	0.08	1.64E-06	0.10	1.97E-06
R12	16	chr16:71997961-72203636	206	235	*DHODH,DHX38,HP,HPR, PKD1L3,PMFBP1,TXNL4B*	chr16_72045090_TG_T	*TXNL4B*	intronic	rs35283911	T/TG	0.292	0.43	3.30E-06	0.46	7.04E-06
R13	19	chr19:38307388-38310647	3.26	3	*YIF1B*	chr19_38307388_C_T	*YIF1B*	intronic	rs79603054	T/C	0.137	2.91	1.45E-05	4.23	2.92E-06

Supplementary Fig. 3. Fisher's exact test computed using healthy individuals of the primary cohort recruited as controls in our study. **(a)** Q-Q plot of Fisher's exact test. **(b)** Manhattan plot of associated variants. **(c)** Summary of significant variants (Fisher's exact test P < 1.0×10^{-5}). Red text indicates the variant with odds ratio (OR) > 1.

Supplementary Fig. 4

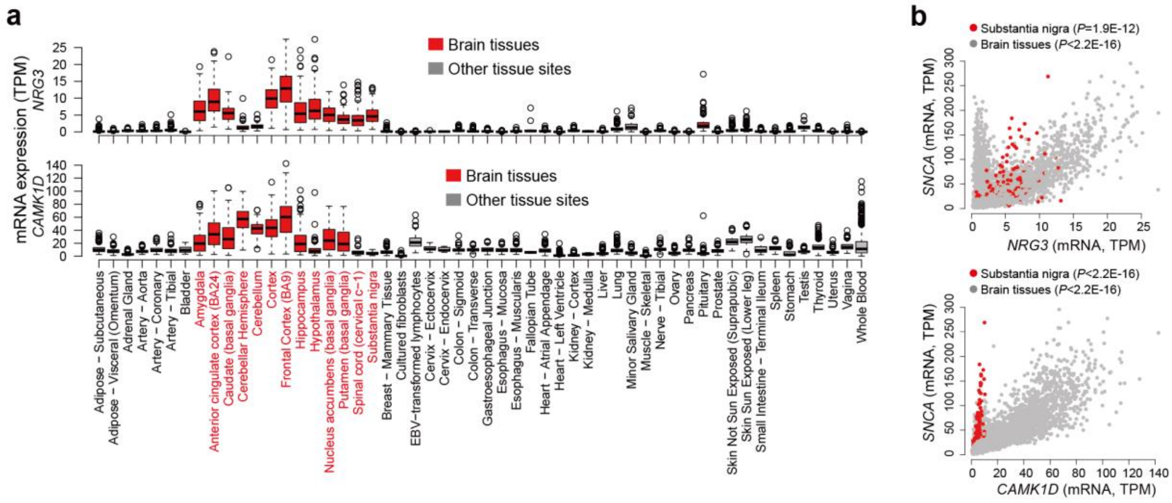

Supplementary Fig. 4. Gene expression data derived from the GTEx project. **(a)** Expression patterns of *NRG3* and *CAMK1D* mRNA across multiple healthy tissues, including the brain. **(b)** Significant correlations between *NRG3*, *CAMK1D*, and *SNCA* mRNA expression (Spearman correlation test).

Supplementary Fig. 5

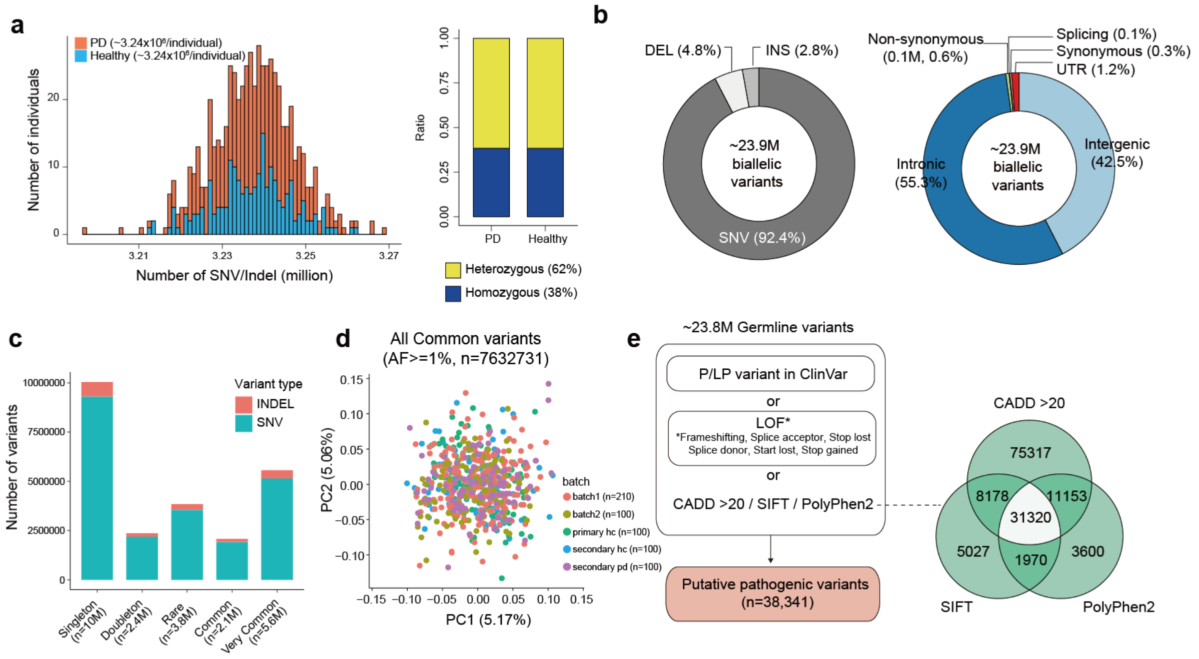

Supplementary Fig. 5. Summary of variant calling from WGS data of 410 PD patients and 200 healthy individuals (primary and secondary cohorts). **(a)** Frequencies of all detected variants. **(b)** Frequencies of different variant types and their genomic locations. **(c)** Number of variants according to allele frequencies. **(d)** PCA plot of variants detected in PD and healthy individuals. **(e)** Detection of pathogenic variants. HC, healthy control; LOF, loss of function; PD, Parkinson's disease; WGS, whole genome sequencing.

Supplementary Fig. 6

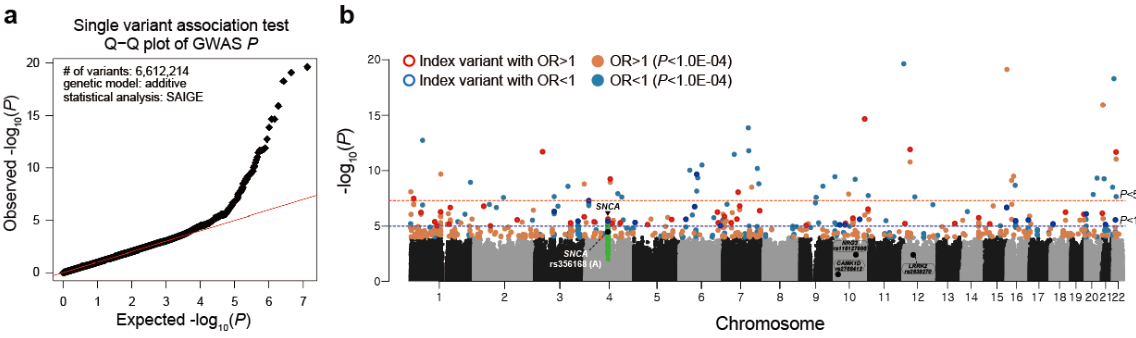

c

No	CHR	RANGE	KB	N	RANGES	Most significant variant in same linkage disequilibrium block									
						Variant identifier	Hugo symbol	Consequence	dbSNP	OR (95% CI)	*P*	A1/A2	MAF	MAF, PD	MAF, KGP
R1	1	chr1:13315231..13373851	58.62	5	*PRAMEF14,PRAMEF15, PRAMEF19*	chr1_13315231_A_C	*PRAMEF15*	intergenic	rs1361212929	1.75 (1.43-2.13)	3.24E-08	C/A	0.206	0.274	0.175
R2	1	chr1:42043437..42089609	46.17	30		chr1_42058403_T_C	*Unknown*	intergenic	rs374316996	1.68 (1.37-2.05)	5.90E-07	C/T	0.197	0.258	0.170
R3	1	chr1:96042484..96086469	43.99	25		chr1_96063919_T_A	*Unknown*	intergenic	rs222886	1.47 (1.25-1.72)	4.06E-06	A/T	0.432	0.500	0.402
R4	1	chr1:104952810..105119772	166.96	27		chr1_105117830_T_A	*Unknown*	intergenic	rs11184440	1.79 (1.38-2.32)	9.60E-06	A/T	0.123	0.165	0.105
R5	1	chr1:119475796..119546311	70.52	91	*HSD3B1*	chr1_119482351_A_C	*Unknown*	intergenic	rs911245	3.03 (1.96-4.66)	5.20E-07	A/C	0.038	0.067	0.026
R6	1	chr1:152937697..152937744	0.05	2		chr1_152937744_G_T	*LINC01527*	intronic	rs1239618819	3.77 (2.11-6.71)	6.75E-06	T/G	0.020	0.039	0.011
R7	1	chr1:157258881..157258949	0.07	3		chr1_157258949_T_C	*Unknown*	intergenic	rs369412326	2.82 (1.91-4.18)	2.10E-07	C/T	0.047	0.080	0.032
R8	2	chr2:40252913..40252914	0.00	2	*SLC8A1,SLC8A1-AS1*	chr2_40252913_G_A	*SLC8A1*	intronic	rs796778837	2.26 (1.66-3.06)	1.62E-07	A/G	0.085	0.128	0.066
R9	2	chr2:66627551..66627557	0.01	2	*LOC100507073*	chr2_66627557_C_G	*LINC01798*	intronic	rs372559935	2.02 (1.48-2.76)	9.60E-06	G/C	0.075	0.110	0.060
R10	2	chr2:172188278..172188291	0.01	2		chr2_172188278_T_C	*AC104088.1*	intronic	rs10167466	1.91 (1.46-2.49)	2.74E-06	C/T	0.098	0.140	0.079
R11	3	chr3:29997305..29997478	0.17	2	*RBMS3*	chr3_29997478_G_T	*RBMS3*	intronic	rs138409334	2.35 (1.86-2.99)	1.97E-12	T/G	0.133	0.207	0.100
R12	3	chr3:75731525..75775681	44.16	5	*MIR4273,ZNF717*	chr3_75740820_G_A	*ZNF717*	intronic	rs1963442	0.56 (0.45-0.7)	4.81E-07	G/A	0.181	0.127	0.205
R13	3	chr3:140237052..140308358	71.31	64	*CLSTN2*	chr3_140256092_T_C	*CLSTN2*	intronic	rs6789431	3.03 (1.88-4.89)	5.21E-06	C/T	0.030	0.054	0.020
R14	3	chr3:169674535..169751413	76.88	60		chr3_169740411_C_T	*Unknown*	intergenic	rs11718668	0.67 (0.56-0.79)	3.61E-06	T/C	0.341	0.276	0.370
R15	3	chr3:194180516..194180517	0.00	2		chr3_194180516_T_G	*Unknown*	intergenic	rs13082179	1.6 (1.32-1.93)	1.47E-06	G/T	0.223	0.284	0.195
R16	4	chr4:14173379..14173390	0.01	3		chr4_14173390_G_A	*AC092546.1*	intronic	rs140342132	0.35 (0.24-0.51)	5.06E-08	G/A	0.054	0.018	0.070
R17	4	chr4:35299944..35329145	29.20	112		chr4_35325334_T_C	*Unknown*	intergenic	rs368671814	2.46 (1.68-3.61)	4.09E-06	C/T	0.046	0.076	0.033
R18	4	chr4:89691681..89862394	170.71	117	*LOC644248,SNCA*	chr4_89780995_G_A	*SNCA*	intronic	rs7655611	0.67 (0.57-0.79)	3.55E-06	G/A	0.425	0.359	0.455
R19	4	chr4:89781878..89924580	142.70	74	*LOC644248,MMRN1,SNCA*	chr4_89786752_C_T	*SNCA*	intronic	rs3775438	1.53 (1.28-1.82)	2.32E-06	T/C	0.354	0.419	0.324
R20	4	chr4:98762300..98762301	0.00	2		chr4_98762300_T_C	*Unknown*	intergenic	rs566996175	5.11 (3.05-8.56)	5.64E-10	C/T	0.029	0.060	0.016
R21	4	chr4:106217818..106217840	0.02	2	*TBCK*	chr4_106217840_A_C	*TBCK*	intronic	rs12644454	1.75 (1.37-2.23)	5.70E-06	C/A	0.123	0.169	0.103
R22	4	chr4:138875404..138884796	9.39	5		chr4_138884796_C_T	*AC109927.1*	intronic	rs578121085	2.09 (1.52-2.88)	5.02E-06	T/C	0.071	0.105	0.055
R23	5	chr5:6575805..6575813	0.01	2		chr5_6575813_T_C	*Unknown*	intergenic	rs4318747	0.37 (0.24-0.56)	4.76E-06	T/C	0.041	0.015	0.052
R24	5	chr5:55642872..55712493	69.62	10	*SLC38A9*	chr5_55651232_G_A	*SLC38A9*	intronic	rs111615640	1.44 (1.23-1.7)	7.38E-06	A/G	0.441	0.509	0.411
R25	5	chr5:150675603..150756762	81.16	4	*DCTN4,MYOZ3,RBM22*	chr5_150675603_C_T	*MYOZ3*	intronic	rs147339561	2.59 (1.74-3.85)	2.53E-06	T/C	0.045	0.074	0.032
R26	6	chr6:25708975..25708995	0.02	2		chr6_25708995_C_T	*Unknown*	intergenic	rs6456691	0.45 (0.32-0.63)	2.42E-06	C/T	0.072	0.038	0.087
R27	6	chr6:60232615..60237912	5.30	2		chr6_60232615_T_C	*Unknown*	intergenic	rs1244077482	0.48 (0.36-0.63)	1.78E-07	T/C	0.111	0.067	0.131
R28	6	chr6:68288577..68289899	1.32	2		chr6_68289899_G_A	*LINC02549*	intronic	rs377680022	0.32 (0.22-0.45)	2.13E-10	A/G	0.057	0.012	0.078
R29	6	chr6:150021407..150021466	0.06	2	*RAET1L*	chr6_150021466_T_A	*RAET1L*	intronic	rs13208269	1.69 (1.38-2.08)	5.47E-07	A/T	0.189	0.248	0.162
R30	6	chr6:153206327..153208312	1.99	2		chr6_153208312_A_G	*Unknown*	intergenic	rs138111223	3.48 (2.04-5.95)	5.04E-06	G/A	0.024	0.045	0.015
R31	6	chr6:161560502..161563662	3.16	5	*PARK2*	chr6_161563345_A_G	*PRKN*	intronic	rs6900355	0.55 (0.42-0.71)	9.18E-06	G/A	0.119	0.079	0.137
R32	7	chr7:6895839..6905518	9.68	19		chr7_6905518_C_T	*AC079804.2*	intergenic	rs148378354	1.6 (1.32-1.93)	1.37E-06	T/C	0.250	0.312	0.223
R33	7	chr7:33839753..33861055	21.30	4		chr7_33861055_C_T	*BBS9*	intronic	rs1421350	1.66 (1.34-2.07)	4.98E-06	C/T	0.165	0.216	0.143
R34	7	chr7:56363677..56363753	0.08	2		chr7_56363753_T_C	*CICP8*	intronic	rs184654394	2.19 (1.58-3.04)	2.36E-06	C/T	0.070	0.105	0.055
R35	7	chr7:60912312..61268506	356.20	156		chr7_61075969_C_G	*Unknown*	intergenic	rs9719559	2.09 (1.63-2.69)	8.68E-09	G/C	0.152	0.207	0.127
R36	7	chr7:67121955..67225790	103.84	5	*TYW1*	chr7_67121955_A_G	*TYW1*	intronic	rs11761332	3.63 (2.24-5.89)	1.55E-07	G/A	0.031	0.059	0.019
R37	7	chr7:146134208..146134210	0.00	2	*CNTNAP2*	chr7_146134210_T_C	*CNTNAP2*	intronic	rs531300297	2.2 (1.62-2.98)	4.06E-07	C/T	0.074	0.115	0.056
R38	9	chr9:25073331..25076312	2.98	4		chr9_25073331_C_G	*Unknown*	intergenic	rs11525924	1.88 (1.42-2.48)	8.61E-06	G/C	0.093	0.132	0.076
R39	10	chr10:19510461..19510462	0.00	2	*MALRD1*	chr10_19510461_C_T	*MALRD1*	intronic	rs117623355	0.56 (0.43-0.72)	7.74E-06	T/C	0.129	0.088	0.148
R40	10	chr10:39489490..39489515	0.03	2		chr10_39489515_G_C	*Unknown*	intergenic	rs1354376777	0.34 (0.21-0.55)	7.76E-06	C/G	0.034	0.011	0.045
R41	10	chr10:41713422..41852883	139.46	6		chr10_41754144_T_G	*Unknown*	intergenic	rs12252224	4.08 (2.22-7.48)	5.54E-06	G/T	0.030	0.049	0.021
R42	10	chr10:71160062..71221484	61.42	11	*UNC5B,UNC5B-AS1*	chr10_71207168_C_T	*Unknown*	intergenic	rs10999730	2.08 (1.54-2.81)	1.95E-06	T/C	0.084	0.122	0.067
R43	10	chr10:97700073..97700074	0.00	2		chr10_97700073_T_C	*Unknown*	intergenic	rs564980123	0.46 (0.33-0.63)	2.46E-06	C/T	0.074	0.039	0.090
R44	10	chr10:117459638..117459666	0.03	4		chr10_117459665_C_A	*AC005871.2*	intronic	rs371244996	3.49 (2.56-4.75)	2.17E-15	A/C	0.074	0.140	0.044
R45	10	chr10:130652071..130652077	0.01	2		chr10_130652071_C_T	*Unknown*	intergenic	rs1169097739	2.2 (1.63-2.97)	2.96E-07	T/C	0.082	0.123	0.063
R46	12	chr12:7814248..7814250	0.00	2	*SLC2A14*	chr12_7814248_A_G	*SLC2A14*	UTR	rs11055940	2.37 (1.63-3.45)	6.00E-06	G/A	0.049	0.079	0.036
R47	12	chr12:27942725..27942735	0.01	2		chr12_27942735_A_T	*Unknown*	intergenic	rs879280320	3.82 (2.64-5.53)	1.23E-12	T/A	0.060	0.109	0.038
R48	13	chr13:86869529..87073930	204.40	8		chr13_86869529_C_T	*Unknown*	intergenic	rs79100337	4.02 (2.2-7.33)	5.90E-06	T/C	0.020	0.039	0.012
R49	14	chr14:82769077..82792251	23.18	6		chr14_82769077_G_T	*Unknown*	intergenic	rs1242558	1.64 (1.34-2.01)	1.77E-06	T/G	0.227	0.283	0.201
R50	15	chr15:36286610..36286656	0.05	2		chr15_36286656_A_G	*Unknown*	intergenic	rs12909572	1.86 (1.42-2.43)	6.40E-06	G/A	0.107	0.148	0.088
R51	16	chr16:1255825..1255832	0.01	4		chr16_1255830_C_T	*TPSD1*	intergenic	rs199666513	0.37 (0.25-0.54)	2.06E-07	C/T	0.056	0.022	0.071
R52	16	chr16:36336981..36337056	0.08	5		chr16_36336981_C_T	*Unknown*	intergenic	rs1156413703	0.48 (0.35-0.65)	3.33E-06	T/C	0.088	0.051	0.104
R53	16	chr16:86647014..86658086	11.07	14		chr16_86647014_G_C	*AC009154.1*	intronic	rs300031	0.65 (0.54-0.78)	2.81E-06	G/C	0.287	0.224	0.314
R54	18	chr18:10653117..10653159	0.04	2		chr18_10653159_A_G	*Unknown*	intergenic	rs77421009	0.37 (0.24-0.57)	6.39E-06	A/G	0.041	0.016	0.053
R55	18	chr18:59985704..59985750	0.05	4		chr18_59985750_G_A	*Unknown*	intergenic	rs190690616	3.99 (2.32-6.87)	5.56E-07	A/G	0.025	0.048	0.014
R56	19	chr19:51909215..51949576	40.36	5	*HCCAT3,ZNF613*	chr19_51909215_G_T	*ZNF649*	intergenic	rs148784679	3.36 (2.07-5.44)	8.52E-07	T/G	0.031	0.056	0.020
R57	20	chr20:2616914..2647525	30.61	32	*TMC2*	chr20_2628927_C_T	*TMC2*	intronic	rs6037150	0.59 (0.48-0.73)	7.99E-07	C/T	0.220	0.164	0.246
R58	21	chr21:7240819..7275791	34.97	2		chr21_7240819_C_T	*Unknown*	intergenic	rs1485751715	2.25 (1.63-3.1)	6.98E-07	T/C	0.081	0.120	0.064
R59	21	chr21:19124546..19298896	174.35	149		chr21_19227666_T_C	*Unknown*	intergenic	rs73206504	0.65 (0.54-0.79)	7.15E-06	C/T	0.266	0.209	0.292
R60	22	chr22:22714738..22746199	31.46	85		chr22_22716135_A_G	*IGLV3-21*	intergenic	rs9620160	0.38 (0.25-0.57)	2.77E-06	G/A	0.044	0.016	0.057
R61	22	chr22:25259718..25259795	0.08	4		chr22_25259791_T_A	*Unknown*	intergenic	rs4049514	5.4 (3.38-8.65)	2.10E-12	A/T	0.035	0.074	0.018

Supplementary Fig. 6. Comparison between AMC, PD, and KGP control. **(a, b)** Common SNVs detected in AMC, PD, and KGP control following application of logistic mixed models to detect risk SNVs. **(c)** Top SNVs most significantly associated with PD. AMC, Asan Medical Center; KGP, Korean Genome Project; PD, Parkinson's disease; SNV, single nucleotide variants.

Supplementary Fig. 7

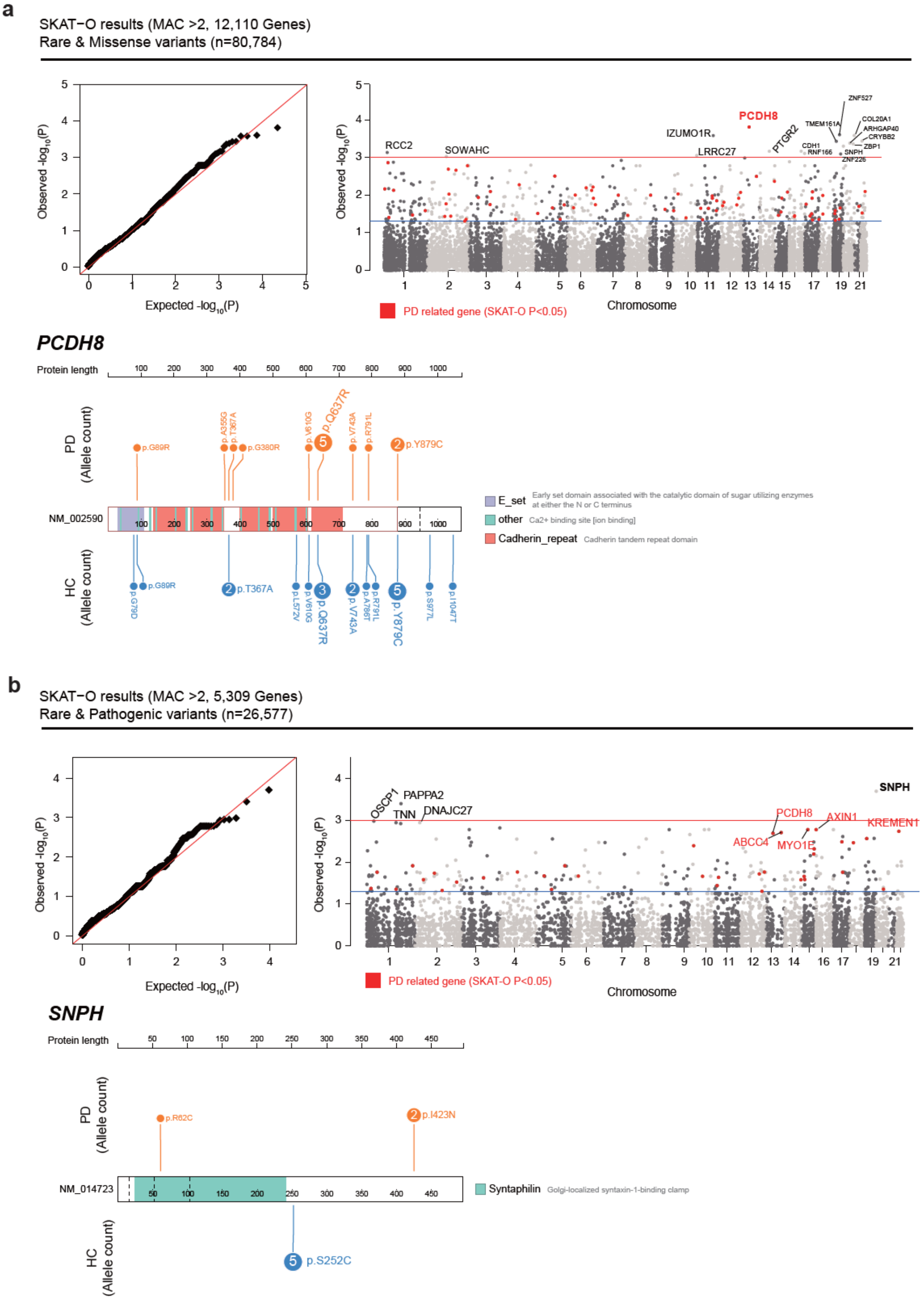

Supplementary Fig. 7. Rare variants associated with PD. **(a)** Genome wide, gene-based SKAT-O analysis of 80 784 rare missense variants and lollipop plot of missense variants in *PCDH8*. **(b)** Genome wide, gene-based SKAT-O analysis of 26,577 rare pathogenic variants and lollipop plot of pathogenic variants in *SNPH*. HC, healthy controls; PD, Parkinson’s disease; SKAT-O, sequence kernel association-optimized.

Supplementary Fig. 8

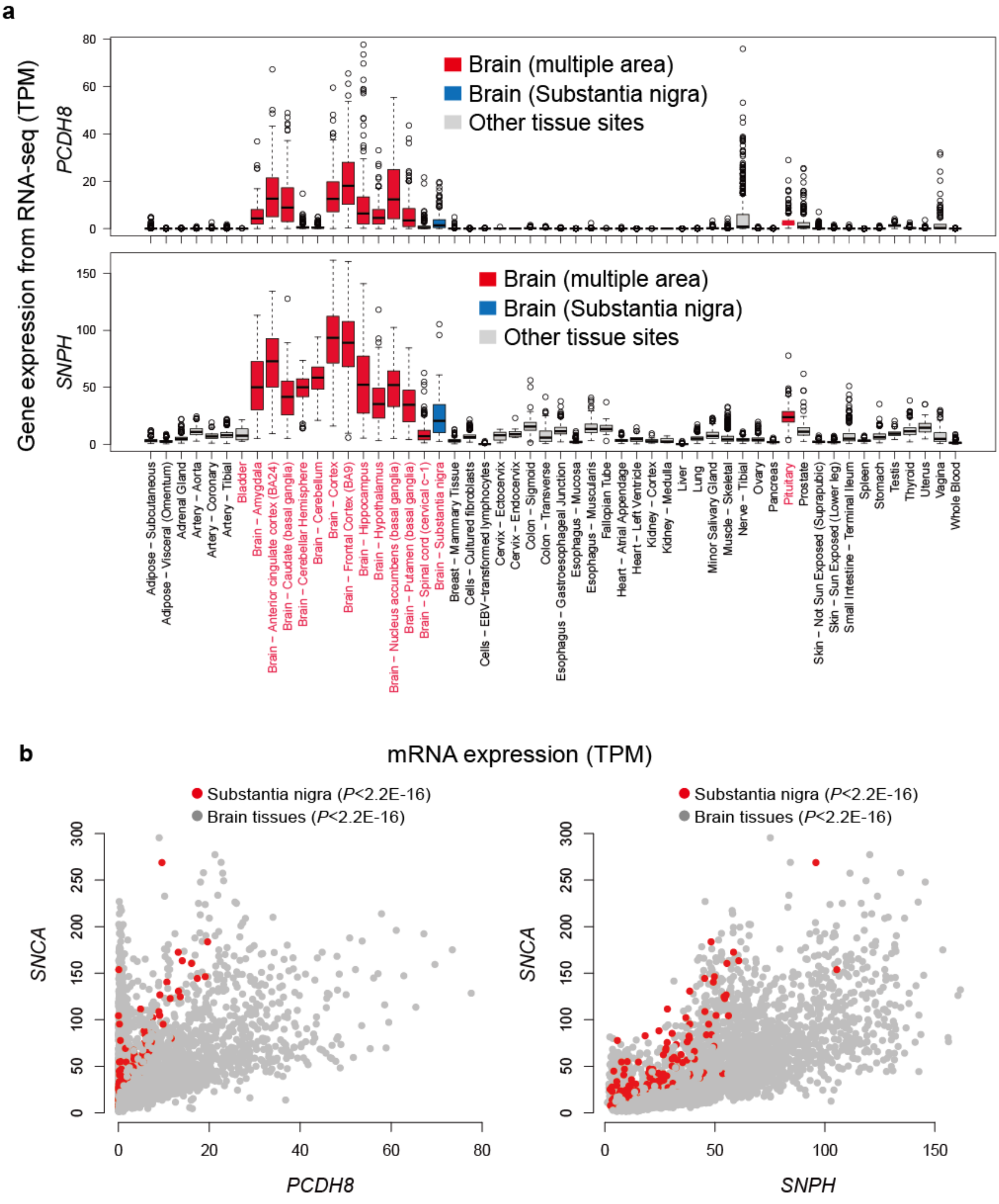

Supplementary Fig. 8. Expression patterns of *PCDH8* and *SNPH* genes in healthy tissues derived from the GTEx dataset. **(a)** Brain tissue-specific expression of *PCDH8* and *SNPH* were identified as the top rare missense or pathogenic variants associated with Parkinson's disease. **(b)** Significant correlations between mRNA expression of *PCDH8* and *SNPH* with *SNCA* in all brain tissues as well as substantia nigra tissues (Spearman correlation test).

Supplementary Fig. 9

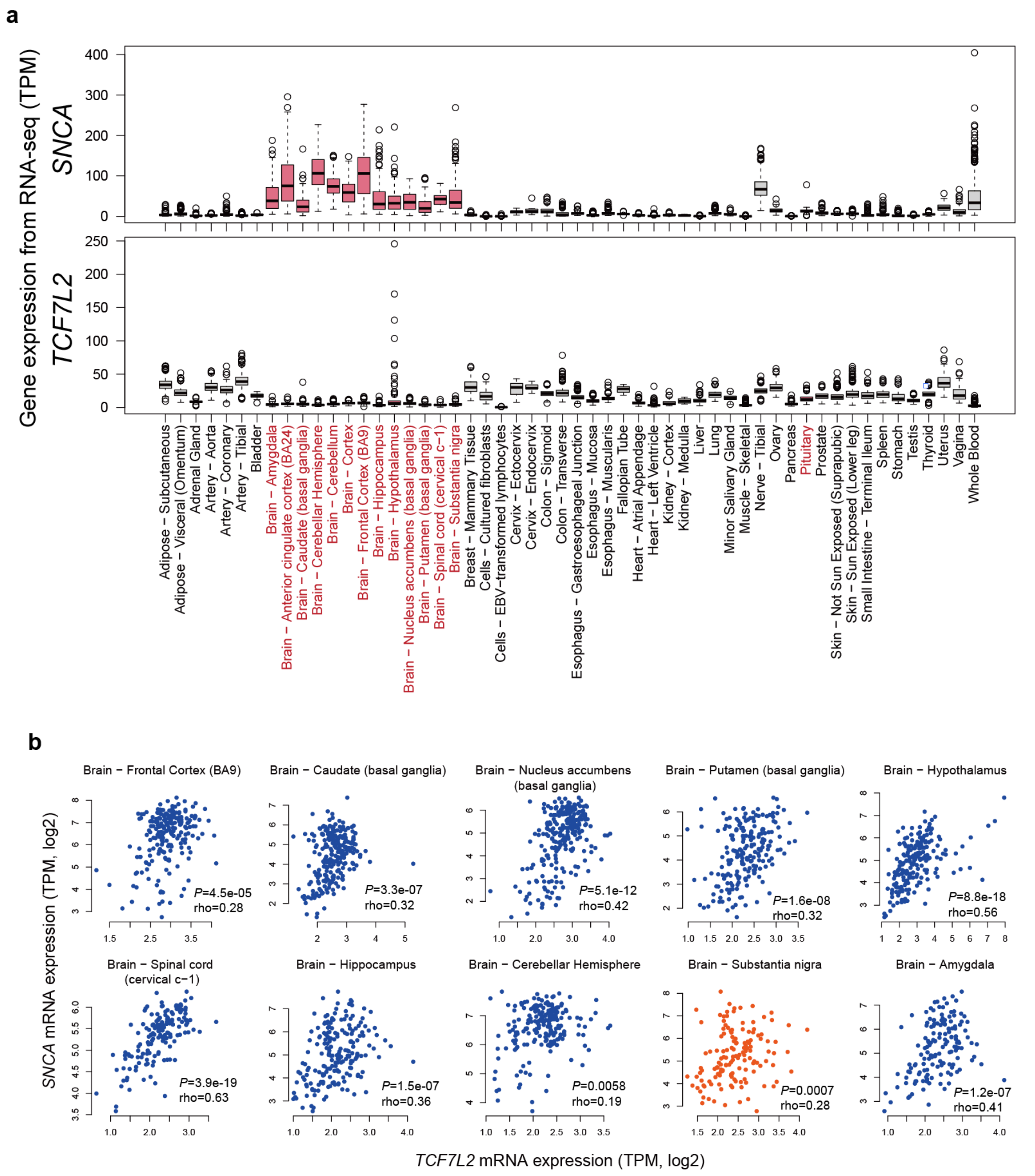

Supplementary Fig. 9. Expression of *SNCA* and *TCF7L2* genes in multiple healthy human tissues. **(a)** *SNCA* gene showed brain tissue-specific expression patterns, whereas *TCFL5* was expressed in multiple tissues. **(b)** Significant correlations between *SNCA* and *TCF7L2* mRNA expression in various brain tissues (Spearman correlation test). Analysis based on data from the GTEx database.

Supplementary Fig. 10

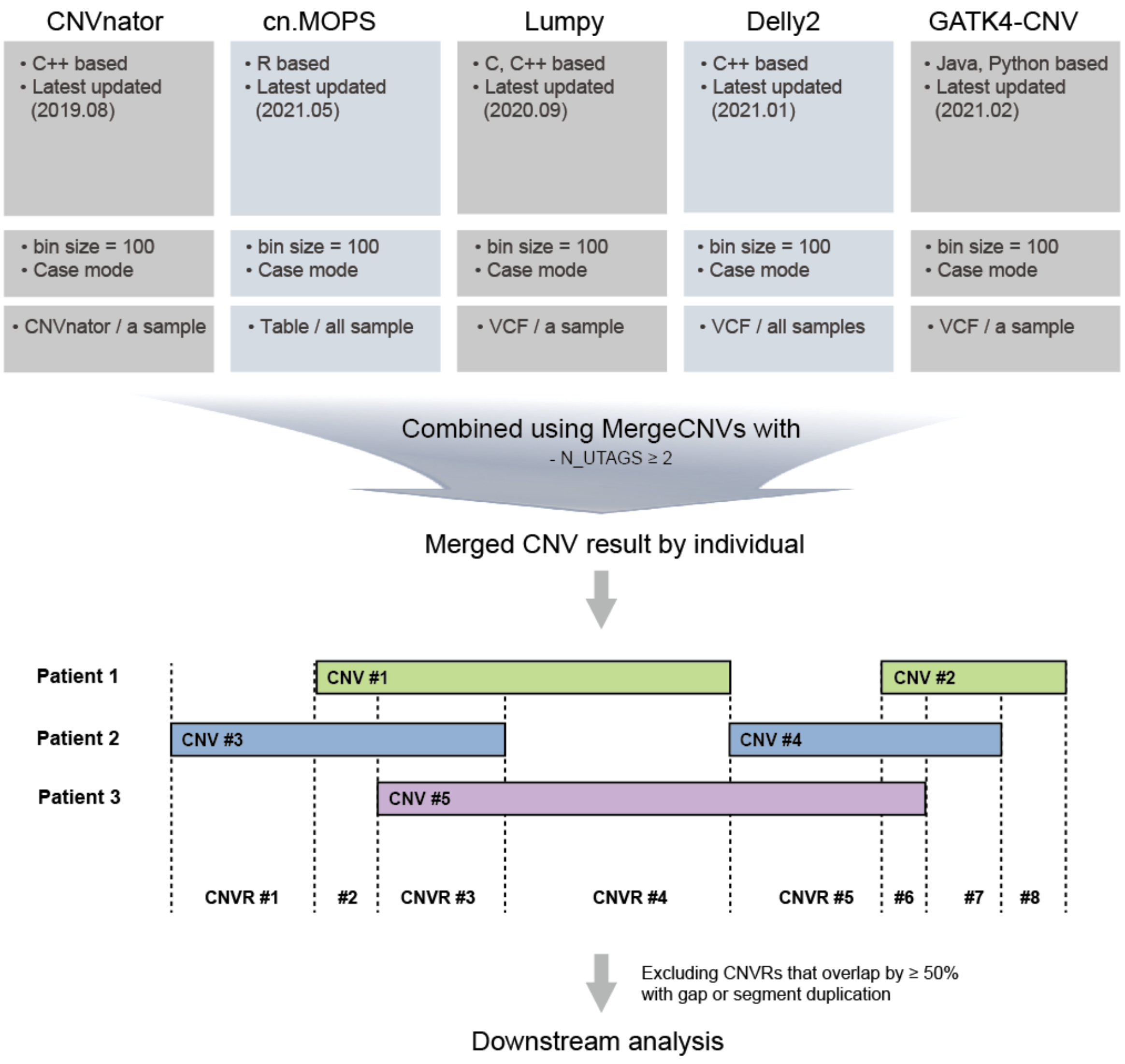

Supplementary Fig. 10. Detection of copy number variations (CNVs) using five different algorithms.

Supplementary Fig. 11

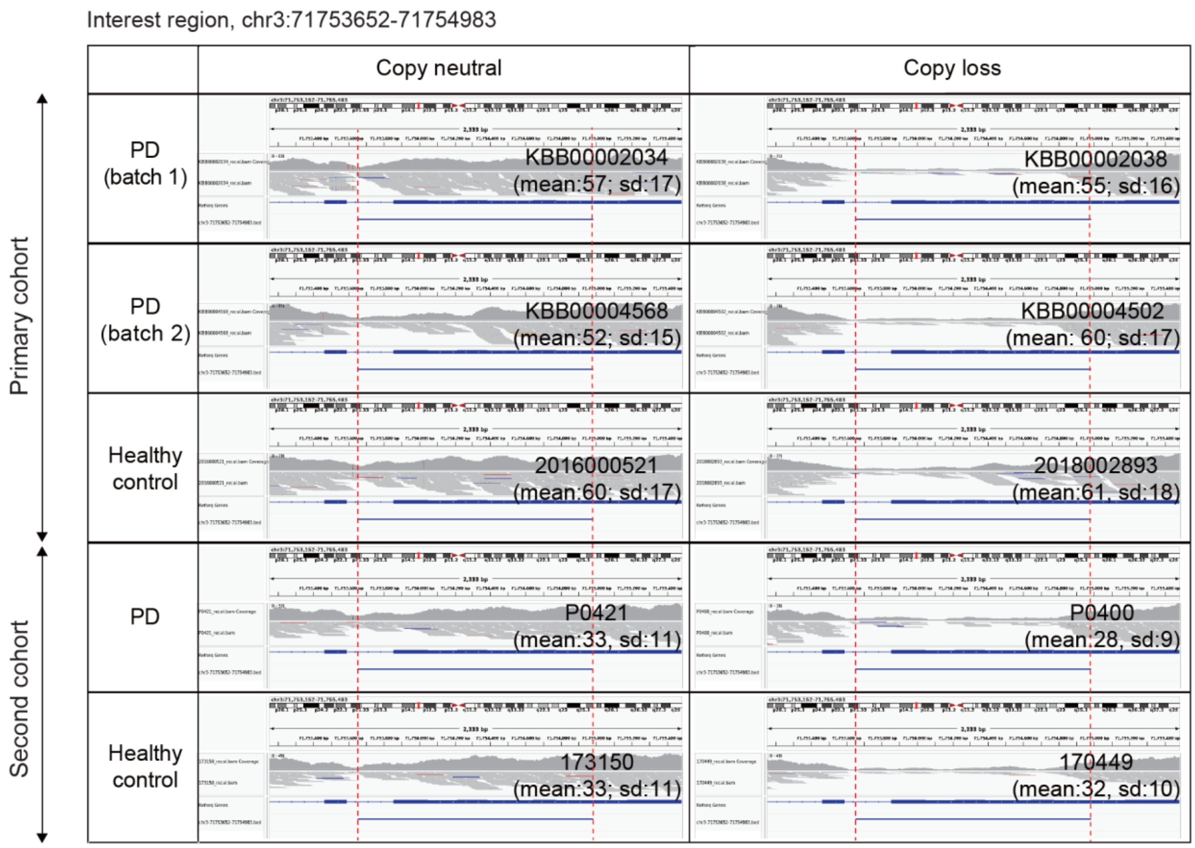

Supplementary Fig. 11. Representative images of copy number deleted regions on chr3 using Integrative Genomics Viewer (IGV).

Supplementary Fig. 12

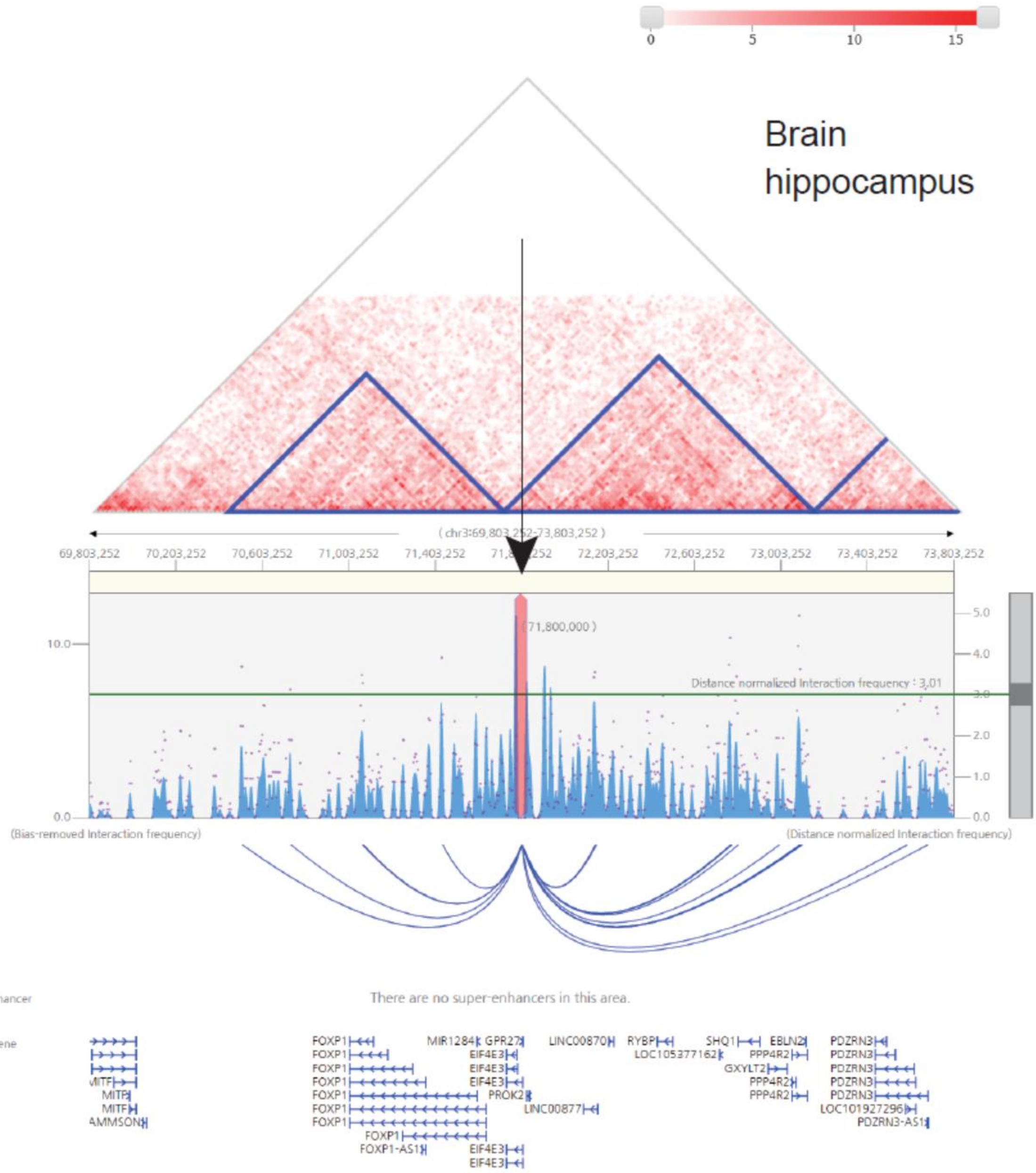

Supplementary Fig. 12. Interaction of the deleted region (chr3:71802803–71804134, hg19) with other genomic regions.

Supplementary Fig. 13

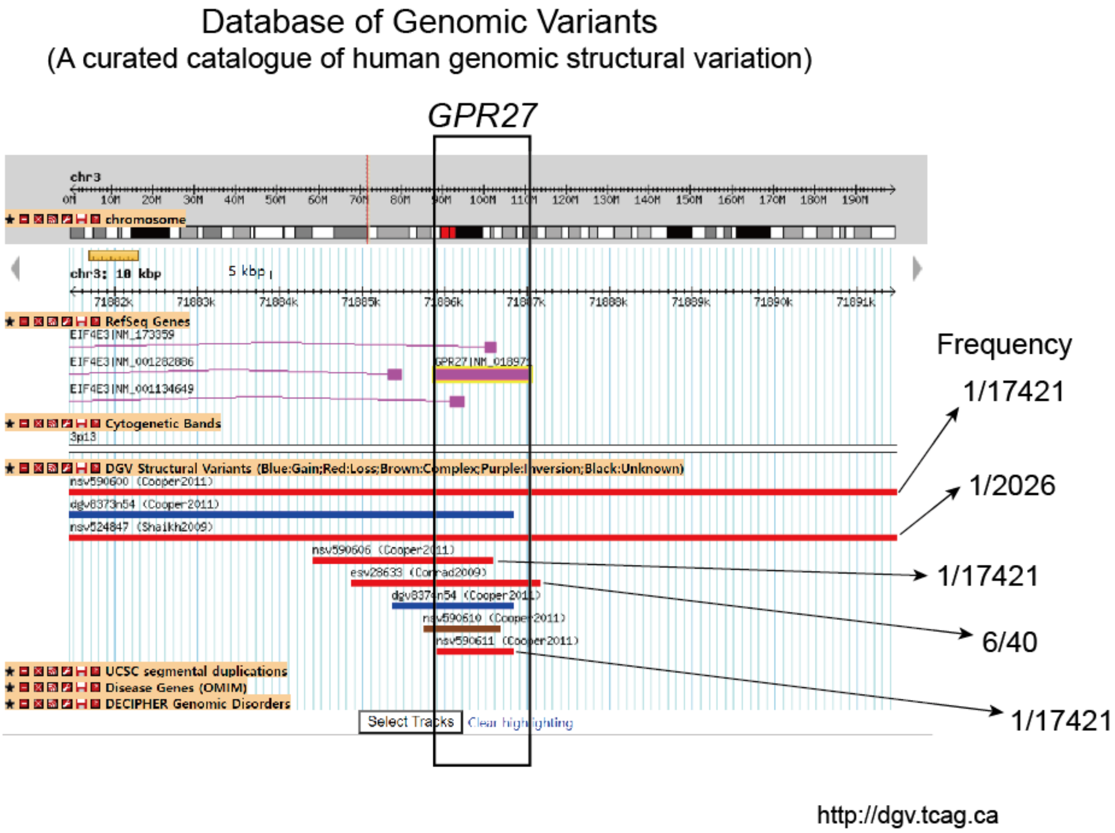

Supplementary Fig. 13. Copy number variation in *GPR27* in the database of human genomic structural variation.

Supplementary Fig. 14

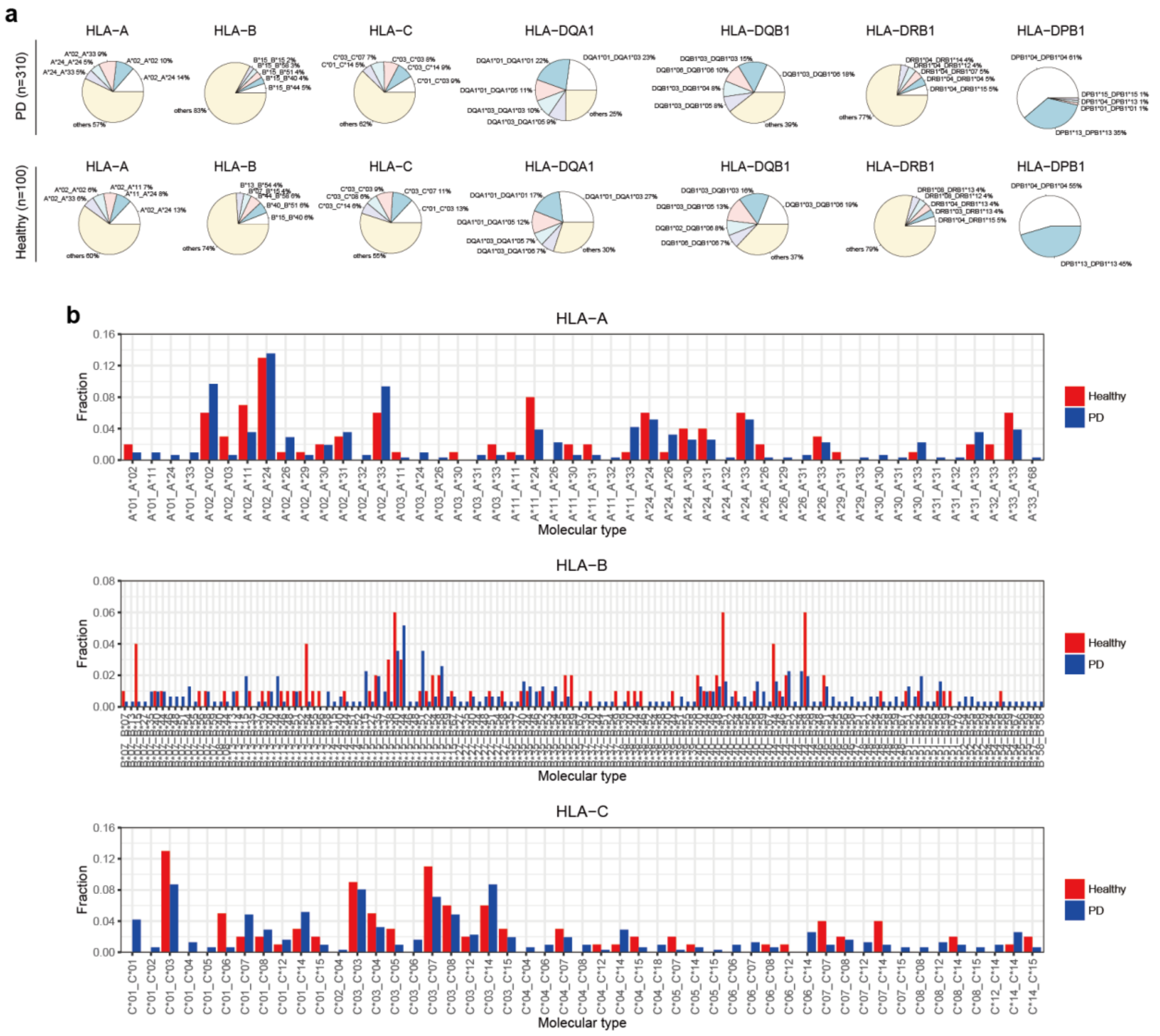

Supplementary Fig. 14. Summary of HLA molecular profiles in 410 individuals. **(a)** Frequencies of HLA molecular types in 310 PD patients and 100 healthy controls. **(b)** No significant differences in frequencies were observed between PD and healthy controls (Fisher's exact test FDR q > 0.05). HLA, human leukocyte antigen; PD, Parkinson's disease.

Supplementary Table 1. Clinical information of 410 individuals (primary cohort).

Subject no.	Sample barcode	Sex	Age at sampling	Age at onset	Group	Batch	Subject no	Sample barcode	Sex	Age at sampling	Age at onset	Group	Batch
N0001	2016000051	M	64	NA	hc	healthy control	P0106	KBB00002064	F	57	42	pd	batch 1
N0002	2016000106	M	72	NA	hc	healthy control	P0107	KBB00002065	M	66	59	pd	batch 1
N0003	2016000209	F	69	NA	hc	healthy control	P0108	KBB00002066	M	72	61	pd	batch 1
N0004	2016000210	F	68	NA	hc	healthy control	P0109	KBB00002067	F	50	47	pd	batch 1
N0005	2016000224	F	57	NA	hc	healthy control	P0110	KBB00002068	F	68	66	pd	batch 1
N0006	2016000310	F	62	NA	hc	healthy control	P0111	KBB00002069	F	73	65	pd	batch 1
N0007	2016000423	F	64	NA	hc	healthy control	P0112	KBB00002070	M	80	75	pd	batch 1
N0008	2016000481	M	70	NA	hc	healthy control	P0113	KBB00002071	F	65	51	pd	batch 1
N0009	2016000521	M	69	NA	hc	healthy control	P0114	KBB00002072	F	70	42	pd	batch 1
N0010	2016001038	F	57	NA	hc	healthy control	P0115	KBB00002073	F	59	55	pd	batch 1
N0011	2016001071	M	63	NA	hc	healthy control	P0116	KBB00002074	F	76	68	pd	batch 1
N0012	2017000071	M	72	NA	hc	healthy control	P0117	KBB00002075	F	63	55	pd	batch 1
N0013	2017000126	F	64	NA	hc	healthy control	P0118	KBB00002076	M	73	57	pd	batch 1
N0014	2017000132	F	59	NA	hc	healthy control	P0119	KBB00002077	M	77	72	pd	batch 1
N0015	2017000152	M	59	NA	hc	healthy control	P0120	KBB00002078	F	64	52	pd	batch 1
N0016	2017000327	F	60	NA	hc	healthy control	P0121	KBB00002079	F	70	67	pd	batch 1
N0017	2017000361	M	68	NA	hc	healthy control	P0122	KBB00002080	F	69	54	pd	batch 1
N0018	2017000433	M	61	NA	hc	healthy control	P0123	KBB00002081	F	61	56	pd	batch 1
N0019	2017000460	M	67	NA	hc	healthy control	P0124	KBB00002082	F	65	61	pd	batch 1
N0020	2017000474	F	62	NA	hc	healthy control	P0125	KBB00002083	F	51	41	pd	batch 1
N0021	2017000546	F	58	NA	hc	healthy control	P0126	KBB00002084	F	69	63	pd	batch 1
N0022	2017000585	M	65	NA	hc	healthy control	P0127	KBB00002085	M	79	65	pd	batch 1
N0023	2017000628	F	61	NA	hc	healthy control	P0128	KBB00002086	M	73	68	pd	batch 1
N0024	2017000686	F	63	NA	hc	healthy control	P0129	KBB00002087	F	56	54	pd	batch 1
N0025	2017000692	F	62	NA	hc	healthy control	P0130	KBB00002088	M	60	54	pd	batch 1
N0026	2017000958	M	63	NA	hc	healthy control	P0131	KBB00002089	F	84	82	pd	batch 1
N0027	2017001086	F	58	NA	hc	healthy control	P0132	KBB00002090	M	70	65	pd	batch 1
N0028	2017001172	M	70	NA	hc	healthy control	P0133	KBB00002091	M	59	59	pd	batch 1
N0029	2017001302	M	58	NA	hc	healthy control	P0134	KBB00002092	F	66	59	pd	batch 1
N0030	2017001383	M	66	NA	hc	healthy control	P0135	KBB00002093	F	70	63	pd	batch 1
N0031	2017001582	F	65	NA	hc	healthy control	P0136	KBB00002094	M	55	45	pd	batch 1
N0032	2017001722	F	63	NA	hc	healthy control	P0137	KBB00002095	F	50	41	pd	batch 1
N0033	2017001813	M	70	NA	hc	healthy control	P0138	KBB00002096	M	60	52	pd	batch 1
N0034	2017001871	F	65	NA	hc	healthy control	P0139	KBB00002097	M	64	56	pd	batch 1
N0035	2017001897	F	64	NA	hc	healthy control	P0140	KBB00002098	F	65	61	pd	batch 1
N0036	2017001932	F	54	NA	hc	healthy control	P0141	KBB00002099	M	58	54	pd	batch 1
N0037	2017001970	F	59	NA	hc	healthy control	P0142	KBB00002100	M	71	69	pd	batch 1
N0038	2017002104	F	64	NA	hc	healthy control	P0143	KBB00002101	M	59	55	pd	batch 1
N0039	2017002191	M	65	NA	hc	healthy control	P0144	KBB00002102	F	68	64	pd	batch 1
N0040	2017002217	F	61	NA	hc	healthy control	P0145	KBB00002103	F	70	66	pd	batch 1
N0041	2017002240	F	57	NA	hc	healthy control	P0146	KBB00002104	M	54	42	pd	batch 1
N0042	2017002275	M	66	NA	hc	healthy control	P0147	KBB00002105	M	66	57	pd	batch 1
N0043	2017002280	F	63	NA	hc	healthy control	P0148	KBB00002106	F	68	57	pd	batch 1
N0044	2017002343	F	58	NA	hc	healthy control	P0149	KBB00002107	F	54	51	pd	batch 1
N0045	2017002346	M	76	NA	hc	healthy control	P0150	KBB00002108	M	78	76	pd	batch 1
N0046	2017002445	F	59	NA	hc	healthy control	P0151	KBB00002109	F	52	45	pd	batch 1
N0047	2017002478	M	59	NA	hc	healthy control	P0152	KBB00002110	M	65	57	pd	batch 1
N0048	2017002550	M	70	NA	hc	healthy control	P0153	KBB00002111	F	54	51	pd	batch 1
N0049	2017002583	M	58	NA	hc	healthy control	P0154	KBB00002112	F	61	53	pd	batch 1
N0050	2017002621	M	60	NA	hc	healthy control	P0155	KBB00002113	F	56	46	pd	batch 1
N0051	2017002633	M	68	NA	hc	healthy control	P0156	KBB00002114	F	76	67	pd	batch 1
N0052	2017002635	F	62	NA	hc	healthy control	P0157	KBB00002115	F	66	59	pd	batch 1
N0053	2017002840	M	55	NA	hc	healthy control	P0158	KBB00002116	F	72	64	pd	batch 1
N0054	2017002875	F	63	NA	hc	healthy control	P0159	KBB00002117	F	64	61	pd	batch 1
N0055	2017002882	F	63	NA	hc	healthy control	P0160	KBB00002118	M	69	57	pd	batch 1
N0056	2017002957	F	59	NA	hc	healthy control	P0161	KBB00002119	F	74	61	pd	batch 1
N0057	2017003005	M	64	NA	hc	healthy control	P0162	KBB00002120	F	75	72	pd	batch 1
N0058	2017003057	M	58	NA	hc	healthy control	P0163	KBB00002121	M	71	65	pd	batch 1
N0059	2017003162	F	61	NA	hc	healthy control	P0164	KBB00002122	F	72	59	pd	batch 1
N0060	2018000134	M	66	NA	hc	healthy control	P0165	KBB00002123	F	70	64	pd	batch 1
N0061	2018000375	M	68	NA	hc	healthy control	P0166	KBB00002124	F	55	42	pd	batch 1
N0062	2018000532	M	70	NA	hc	healthy control	P0167	KBB00002125	F	71	68	pd	batch 1
N0063	2018000568	M	65	NA	hc	healthy control	P0168	KBB00002126	F	70	62	pd	batch 1
N0064	2018000571	M	74	NA	hc	healthy control	P0169	KBB00002127	M	70	63	pd	batch 1
N0065	2018000614	F	62	NA	hc	healthy control	P0170	KBB00002128	M	68	61	pd	batch 1
N0066	2018000637	M	71	NA	hc	healthy control	P0171	KBB00002129	M	63	61	pd	batch 1
N0067	2018000684	M	69	NA	hc	healthy control	P0172	KBB00002130	M	71	67	pd	batch 1
N0068	2018000749	F	61	NA	hc	healthy control	P0173	KBB00002131	M	66	65	pd	batch 1
N0069	2018000790	M	71	NA	hc	healthy control	P0174	KBB00002132	M	59	52	pd	batch 1
N0070	2018000976	F	66	NA	hc	healthy control	P0175	KBB00002133	M	67	57	pd	batch 1
N0071	2018001046	F	63	NA	hc	healthy control	P0176	KBB00002134	M	59	51	pd	batch 1
N0072	2018001061	F	59	NA	hc	healthy control	P0177	KBB00002135	F	66	63	pd	batch 1
N0073	2018001132	M	70	NA	hc	healthy control	P0178	KBB00002136	M	56	53	pd	batch 1
N0074	2018001164	M	61	NA	hc	healthy control	P0179	KBB00002137	M	68	57	pd	batch 1
N0075	2018001310	F	62	NA	hc	healthy control	P0180	KBB00002138	F	60	49	pd	batch 1
N0076	2018001362	F	60	NA	hc	healthy control	P0181	KBB00002139	M	75	59	pd	batch 1
N0077	2018001380	M	70	NA	hc	healthy control	P0182	KBB00002140	F	62	53	pd	batch 1
N0078	2018001494	F	64	NA	hc	healthy control	P0183	KBB00002141	M	68	64	pd	batch 1
N0079	2018001519	M	64	NA	hc	healthy control	P0184	KBB00002142	M	58	55	pd	batch 1
N0080	2018001526	M	55	NA	hc	healthy control	P0185	KBB00002143	M	66	61	pd	batch 1
N0081	2018001540	M	56	NA	hc	healthy control	P0186	KBB00002144	M	67	62	pd	batch 1
N0082	2018001553	M	81	NA	hc	healthy control	P0187	KBB00002145	F	64	55	pd	batch 1
N0083	2018001564	M	68	NA	hc	healthy control	P0188	KBB00002146	F	76	65	pd	batch 1
N0084	2018001588	M	69	NA	hc	healthy control	P0189	KBB00002147	F	72	65	pd	batch 1
N0085	2018001677	F	60	NA	hc	healthy control	P0190	KBB00002148	M	69	67	pd	batch 1
N0086	2018001744	F	61	NA	hc	healthy control	P0191	KBB00002149	M	71	65	pd	batch 1
N0087	2018001773	M	66	NA	hc	healthy control	P0192	KBB00002150	M	67	65	pd	batch 1
N0088	2018001860	F	61	NA	hc	healthy control	P0193	KBB00002151	M	71	68	pd	batch 1
N0089	2018002002	F	61	NA	hc	healthy control	P0194	KBB00002152	F	42	41	pd	batch 1
N0090	2018002089	F	63	NA	hc	healthy control	P0195	KBB00002153	M	70	64	pd	batch 1
N0091	2018002271	F	62	NA	hc	healthy control	P0196	KBB00002154	F	56	46	pd	batch 1
N0092	2018002370	F	61	NA	hc	healthy control	P0197	KBB00002155	M	64	49	pd	batch 1
N0093	2018002722	F	57	NA	hc	healthy control	P0198	KBB00002156	M	68	51	pd	batch 1
N0094	2018002769	M	72	NA	hc	healthy control	P0199	KBB00002157	M	53	47	pd	batch 1
N0095	2018002879	M	57	NA	hc	healthy control	P0200	KBB00002158	M	71	69	pd	batch 1
N0096	2018002890	M	59	NA	hc	healthy control	P0201	KBB00002159	F	67	66	pd	batch 1
N0097	2018002893	M	55	NA	hc	healthy control	P0202	KBB00002160	M	72	73	pd	batch 1
N0098	2018002987	M	55	NA	hc	healthy control	P0203	KBB00002161	F	72	69	pd	batch 1
N0099	2018003012	F	54	NA	hc	healthy control	P0204	KBB00002162	M	66	65	pd	batch 1
N0100	2018003096	F	63	NA	hc	healthy control	P0205	KBB00002163	M	47	44	pd	batch 1
P0001	KBB00001959	M	49	47	pd	batch 1	P0206	KBB00002164	F	52	52	pd	batch 1
P0002	KBB00001960	M	51	48	pd	batch 1	P0207	KBB00002165	F	75	74	pd	batch 1
P0003	KBB00001961	M	46	45	pd	batch 1	P0208	KBB00002166	M	52	51	pd	batch 1
P0004	KBB00001962	M	80	77	pd	batch 1	P0209	KBB00002167	F	69	69	pd	batch 1
P0005	KBB00001963	M	61	59	pd	batch 1	P0210	KBB00002168	M	70	69	pd	batch 1
P0006	KBB00001964	M	57	55	pd	batch 1	P0211	KBB00003842	F	76	43	pd	batch 2
P0007	KBB00001965	M	46	45	pd	batch 1	P0212	KBB00003843	M	74	71	pd	batch 2
P0008	KBB00001966	F	68	66	pd	batch 1	P0213	KBB00003844	M	49	46	pd	batch 2
P0009	KBB00001967	F	62	59	pd	batch 1	P0214	KBB00003855	M	83	80	pd	batch 2
P0010	KBB00001968	M	71	66	pd	batch 1	P0215	KBB00003856	F	59	55	pd	batch 2
P0011	KBB00001969	M	74	74	pd	batch 1	P0216	KBB00003857	M	75	72	pd	batch 2
P0012	KBB00001970	F	62	61	pd	batch 1	P0217	KBB00003877	F	69	65	pd	batch 2
P0013	KBB00001971	M	43	41	pd	batch 1	P0218	KBB00003885	F	76	73	pd	batch 2
P0014	KBB00001972	M	58	57	pd	batch 1	P0219	KBB00003886	F	58	52	pd	batch 2
P0015	KBB00001973	M	64	62	pd	batch 1	P0220	KBB00003887	F	53	49	pd	batch 2
P0016	KBB00001974	M	53	52	pd	batch 1	P0221	KBB00003909	M	64	65	pd	batch 2
P0017	KBB00001975	M	57	53	pd	batch 1	P0222	KBB00003917	F	65	64	pd	batch 2
P0018	KBB00001976	M	62	62	pd	batch 1	P0223	KBB00003918	F	65	62	pd	batch 2
P0019	KBB00001977	M	59	58	pd	batch 1	P0224	KBB00003933	F	63	61	pd	batch 2
P0020	KBB00001978	F	63	62	pd	batch 1	P0225	KBB00003941	F	48	46	pd	batch 2
P0021	KBB00001979	F	63	63	pd	batch 1	P0226	KBB00003942	F	67	65	pd	batch 2

P0022	KBB00001980	F	73	72	pd	batch 1	P0227	KBB00003943	F	63	59	pd	batch 2
P0023	KBB00001981	F	66	64	pd	batch 1	P0228	KBB00003965	M	80	75	pd	batch 2
P0024	KBB00001982	M	52	50	pd	batch 1	P0229	KBB00003966	F	68	65	pd	batch 2
P0025	KBB00001983	M	72	69	pd	batch 1	P0230	KBB00003967	M	69	66	pd	batch 2
P0026	KBB00001984	M	71	70	pd	batch 1	P0231	KBB00003988	M	79	75	pd	batch 2
P0027	KBB00001985	F	55	52	pd	batch 1	P0232	KBB00003996	M	69	67	pd	batch 2
P0028	KBB00001986	M	55	52	pd	batch 1	P0233	KBB00003997	F	57	55	pd	batch 2
P0029	KBB00001987	M	59	59	pd	batch 1	P0234	KBB00004012	M	60	53	pd	batch 2
P0030	KBB00001988	F	71	67	pd	batch 1	P0236	KBB00004020	M	55	52	pd	batch 2
P0031	KBB00001989	F	79	64	pd	batch 1	P0237	KBB00004028	F	64	62	pd	batch 2
P0032	KBB00001990	M	61	57	pd	batch 1	P0238	KBB00004029	F	64	61	pd	batch 2
P0033	KBB00001991	F	61	56	pd	batch 1	P0239	KBB00004030	M	84	81	pd	batch 2
P0034	KBB00001992	F	61	60	pd	batch 1	P0240	KBB00004052	F	69	64	pd	batch 2
P0035	KBB00001993	M	64	61	pd	batch 1	P0241	KBB00004053	M	64	57	pd	batch 2
P0036	KBB00001994	M	65	62	pd	batch 1	P0242	KBB00004054	F	54	53	pd	batch 2
P0037	KBB00001995	F	74	61	pd	batch 1	P0243	KBB00004055	M	65	59	pd	batch 2
P0038	KBB00001996	M	70	62	pd	batch 1	P0244	KBB00004084	M	68	66	pd	batch 2
P0039	KBB00001997	M	73	73	pd	batch 1	P0246	KBB00004085	F	77	72	pd	batch 2
P0040	KBB00001998	M	68	61	pd	batch 1	P0247	KBB00004086	F	58	55	pd	batch 2
P0041	KBB00001999	F	50	44	pd	batch 1	P0248	KBB00004087	F	51	48	pd	batch 2
P0042	KBB00002000	M	69	66	pd	batch 1	P0249	KBB00004088	F	58	52	pd	batch 2
P0043	KBB00002001	F	56	49	pd	batch 1	P0250	KBB00004124	M	66	60	pd	batch 2
P0044	KBB00002002	M	77	75	pd	batch 1	P0251	KBB00004125	F	79	76	pd	batch 2
P0045	KBB00002003	F	75	66	pd	batch 1	P0252	KBB00004156	F	70	67	pd	batch 2
P0046	KBB00002004	F	85	78	pd	batch 1	P0255	KBB00004157	M	41	38	pd	batch 2
P0047	KBB00002005	M	64	64	pd	batch 1	P0256	KBB00004158	F	48	44	pd	batch 2
P0048	KBB00002006	F	76	66	pd	batch 1	P0257	KBB00004159	M	80	77	pd	batch 2
P0049	KBB00002007	F	64	60	pd	batch 1	P0258	KBB00004160	M	57	53	pd	batch 2
P0050	KBB00002008	F	75	65	pd	batch 1	P0259	KBB00004162	F	74	70	pd	batch 2
P0051	KBB00002009	M	54	50	pd	batch 1	P0261	KBB00004212	F	66	64	pd	batch 2
P0052	KBB00002010	M	66	62	pd	batch 1	P0262	KBB00004220	M	69	69	pd	batch 2
P0053	KBB00002011	F	73	70	pd	batch 1	P0263	KBB00004228	M	64	59	pd	batch 2
P0054	KBB00002012	F	66	63	pd	batch 1	P0264	KBB00004229	F	59	54	pd	batch 2
P0055	KBB00002013	M	75	72	pd	batch 1	P0265	KBB00004230	M	73	71	pd	batch 2
P0056	KBB00002014	F	56	52	pd	batch 1	P0266	KBB00004231	M	54	51	pd	batch 2
P0057	KBB00002015	F	77	68	pd	batch 1	P0267	KBB00004260	F	70	63	pd	batch 2
P0058	KBB00002016	F	76	69	pd	batch 1	P0268	KBB00004261	M	59	56	pd	batch 2
P0059	KBB00002017	M	63	57	pd	batch 1	P0269	KBB00004262	M	74	70	pd	batch 2
P0060	KBB00002018	M	83	70	pd	batch 1	P0270	KBB00004263	F	64	61	pd	batch 2
P0061	KBB00002019	F	73	68	pd	batch 1	P0271	KBB00004264	M	52	47	pd	batch 2
P0062	KBB00002020	M	71	69	pd	batch 1	P0272	KBB00004300	F	63	62	pd	batch 2
P0063	KBB00002021	F	75	60	pd	batch 1	P0273	KBB00004301	M	70	64	pd	batch 2
P0064	KBB00002022	F	67	66	pd	batch 1	P0274	KBB00004302	F	70	67	pd	batch 2
P0065	KBB00002023	M	71	60	pd	batch 1	P0275	KBB00004303	F	79	74	pd	batch 2
P0066	KBB00002024	F	70	62	pd	batch 1	P0276	KBB00004304	F	48	43	pd	batch 2
P0067	KBB00002025	F	63	59	pd	batch 1	P0277	KBB00004305	F	77	71	pd	batch 2
P0068	KBB00002026	M	68	56	pd	batch 1	P0278	KBB00004306	M	65	62	pd	batch 2
P0069	KBB00002027	F	74	67	pd	batch 1	P0279	KBB00004307	F	48	42	pd	batch 2
P0070	KBB00002028	M	42	29	pd	batch 1	P0280	KBB00004308	M	57	46	pd	batch 2
P0071	KBB00002029	F	84	69	pd	batch 1	P0281	KBB00004309	M	51	48	pd	batch 2
P0072	KBB00002030	F	66	55	pd	batch 1	P0282	KBB00004310	M	75	62	pd	batch 2
P0073	KBB00002031	F	83	73	pd	batch 1	P0283	KBB00004388	M	56	53	pd	batch 2
P0074	KBB00002032	M	80	68	pd	batch 1	P0284	KBB00004389	F	67	55	pd	batch 2
P0075	KBB00002033	F	69	59	pd	batch 1	P0285	KBB00004390	F	68	56	pd	batch 2
P0076	KBB00002034	M	72	66	pd	batch 1	P0286	KBB00004391	M	76	74	pd	batch 2
P0077	KBB00002035	M	62	58	pd	batch 1	P0287	KBB00004392	M	61	58	pd	batch 2
P0078	KBB00002036	M	75	71	pd	batch 1	P0288	KBB00004393	M	64	59	pd	batch 2
P0079	KBB00002037	M	72	63	pd	batch 1	P0289	KBB00004394	M	54	50	pd	batch 2
P0080	KBB00002038	F	55	54	pd	batch 1	P0291	KBB00004395	M	61	45	pd	batch 2
P0081	KBB00002039	F	77	64	pd	batch 1	P0292	KBB00004396	F	77	71	pd	batch 2
P0082	KBB00002040	F	60	50	pd	batch 1	P0293	KBB00004397	F	57	53	pd	batch 2
P0083	KBB00002041	M	59	52	pd	batch 1	P0294	KBB00004398	M	76	73	pd	batch 2
P0084	KBB00002042	F	62	57	pd	batch 1	P0295	KBB00004399	F	74	68	pd	batch 2
P0085	KBB00002043	F	57	50	pd	batch 1	P0296	KBB00004400	F	68	66	pd	batch 2
P0086	KBB00002044	M	74	71	pd	batch 1	P0297	KBB00004401	M	63	61	pd	batch 2
P0087	KBB00002045	F	48	41	pd	batch 1	P0298	KBB00004500	F	73	62	pd	batch 2
P0088	KBB00002046	M	76	62	pd	batch 1	P0299	KBB00004501	F	65	58	pd	batch 2
P0089	KBB00002047	F	70	66	pd	batch 1	P0300	KBB00004502	F	58	53	pd	batch 2
P0090	KBB00002048	F	65	62	pd	batch 1	P0301	KBB00004503	F	79	77	pd	batch 2
P0091	KBB00002049	F	57	54	pd	batch 1	P0302	KBB00004504	F	70	62	pd	batch 2
P0092	KBB00002050	F	83	82	pd	batch 1	P0303	KBB00004505	F	73	71	pd	batch 2
P0093	KBB00002051	M	73	62	pd	batch 1	P0304	KBB00004506	M	65	58	pd	batch 2
P0094	KBB00002052	M	73	65	pd	batch 1	P0305	KBB00004507	M	72	72	pd	batch 2
P0095	KBB00002053	F	70	63	pd	batch 1	P0306	KBB00004564	M	45	37	pd	batch 2
P0096	KBB00002054	F	65	58	pd	batch 1	P0307	KBB00004565	F	50	47	pd	batch 2
P0097	KBB00002055	M	56	48	pd	batch 1	P0308	KBB00004566	F	65	58	pd	batch 2
P0098	KBB00002056	F	69	62	pd	batch 1	P0309	KBB00004567	M	61	59	pd	batch 2
P0099	KBB00002057	F	66	61	pd	batch 1	P0310	KBB00004568	F	63	60	pd	batch 2
P0100	KBB00002058	M	70	63	pd	batch 1	P0311	KBB00004604	F	74	66	pd	batch 2
P0101	KBB00002059	M	55	50	pd	batch 1	P0312	KBB00004605	F	65	64	pd	batch 2
P0102	KBB00002060	M	77	64	pd	batch 1	P0313	KBB00004606	M	62	53	pd	batch 2
P0103	KBB00002061	M	69	55	pd	batch 1	P0314	KBB00004607	M	69	68	pd	batch 2
P0104	KBB00002062	F	71	66	pd	batch 1	P0316	KBB00004608	M	71	65	pd	batch 2
P0105	KBB00002063	F	53	50	pd	batch 1	P0317	KBB00004609	F	51	49	pd	batch 2

Supplementary Table 2. Enhancers of ChIP-seq signal at rs35936842 (*TCF7L2*) in multiple tissues.

Group	Mnemonic	H3K4me1	H3K27ac	Group	Mnemonic	H3K4me1	H3K27ac
IMR90	LNG.IMR90	H3K4me1_Enh	H3K27ac_Enh	Brain	BRN.HIPP.MID	H3K4me1_Enh	H3K27ac_Enh
ESC	ESC.WA7	H3K4me1_Enh		Brain	BRN.SUB.NIG	H3K4me1_Enh	H3K27ac_Enh
ESC	ESC.H9	H3K4me1_Enh	H3K27ac_Enh	Brain	BRN.ANT.CAUD	H3K4me1_Enh	H3K27ac_Enh
ESC	ESC.I3	H3K4me1_Enh		Brain	BRN.CING.GYR	H3K4me1_Enh	H3K27ac_Enh
ESC	ESC.HUES6	H3K4me1_Enh	H3K27ac_Enh	Brain	BRN.INF.TMP	H3K4me1_Enh	H3K27ac_Enh
ESC	ESC.HUES48	H3K4me1_Enh	H3K27ac_Enh	Brain	BRN.ANG.GYR	H3K4me1_Enh	H3K27ac_Enh
ESC	ESC.HUES64	H3K4me1_Enh	H3K27ac_Enh	Brain	BRN.DL.PRFRNTL.CRTX	H3K4me1_Enh	H3K27ac_Enh
ESC	ESC.H1	H3K4me1_Enh	H3K27ac_Enh	Brain	BRN.GRM.MTRX	H3K4me1_Enh	
ESC	ESC.4STAR	H3K4me1_Enh		Brain	BRN.FET.F	H3K4me1_Enh	
iPSC	IPSC.20B	H3K4me1_Enh	H3K27ac_Enh	Brain	BRN.FET.M	H3K4me1_Enh	
iPSC	IPSC.18	H3K4me1_Enh	H3K27ac_Enh	Adipose	FAT.ADIP.NUC	H3K4me1_Enh	H3K27ac_Enh
iPSC	IPSC.15b	H3K4me1_Enh		Muscle	MUS.PSOAS	H3K4me1_Enh	H3K27ac_Enh
iPSC	IPSC.DF.6.9	H3K4me1_Enh	H3K27ac_Enh	Muscle	MUS.SKLT.F	H3K4me1_Enh	H3K27ac_Enh
iPSC	IPSC.DF.19.11	H3K4me1_Enh	H3K27ac_Enh	Muscle	MUS.SKLT.M	H3K4me1_Enh	
ES-deriv	ESDR.H1.NEUR.PROG	H3K4me1_Enh	H3K27ac_Enh	Muscle	MUS.TRNK.FET	H3K4me1_Enh	H3K27ac_Enh
ES-deriv	ESDR.H9.NEUR.PROG	H3K4me1_Enh		Muscle	MUS.LEG.FET	H3K4me1_Enh	H3K27ac_Enh
ES-deriv	ESDR.H9.NEUR	H3K4me1_Enh		Heart	HRT.FET	H3K4me1_Enh	
ES-deriv	ESDR.CD56.MESO	H3K4me1_Enh	H3K27ac_Enh	Heart	HRT.ATR.R	H3K4me1_Enh	H3K27ac_Enh
ES-deriv	ESDR.CD56.ECTO	H3K4me1_Enh	H3K27ac_Enh	Heart	HRT.VENT.L	H3K4me1_Enh	H3K27ac_Enh
ES-deriv	ESDR.CD184.ENDO	H3K4me1_Enh	H3K27ac_Enh	Heart	HRT.VNT.R	H3K4me1_Enh	H3K27ac_Enh
ES-deriv	ESDR.H1.BMP4.MESO	H3K4me1_Enh	H3K27ac_Enh	Heart	VAS.AOR	H3K4me1_Enh	H3K27ac_Enh
ES-deriv	ESDR.H1.BMP4.TROP	H3K4me1_Enh	H3K27ac_Enh	Sm. Muscle	GI.DUO.SM.MUS	H3K4me1_Enh	H3K27ac_Enh
ES-deriv	ESDR.H1.MSC	H3K4me1_Enh	H3K27ac_Enh	Sm. Muscle	GI.CLN.SM.MUS	H3K4me1_Enh	H3K27ac_Enh
Blood & T-cell	BLD.PER.MONUC.PC			Sm. Muscle	GI.RECT.SM.MUS	H3K4me1_Enh	H3K27ac_Enh
Blood & T-cell	BLD.CD3.PPC	H3K4me1_Enh		Sm. Muscle	GI.STMC.MUS	H3K4me1_Enh	H3K27ac_Enh
Blood & T-cell	BLD.CD4.CD25I.CD127.TMEMPC			Digestive	GI.STMC.FET	H3K4me1_Enh	H3K27ac_Enh
Blood & T-cell	BLD.CD3.CPC	H3K4me1_Enh		Digestive	GI.S.INT.FET	H3K4me1_Enh	H3K27ac_Enh
Blood & T-cell	BLD.CD4.CD25.CD127M.TREGPC			Digestive	GI.L.INT.FET	H3K4me1_Enh	H3K27ac_Enh
Blood & T-cell	BLD.CD4.CD25M.TPC			Digestive	GI.S.INT	H3K4me1_Enh	H3K27ac_Enh
Blood & T-cell	BLD.CD4.CD25M.CD45RA.NPC			Digestive	GI.CLN.SIG	H3K4me1_Enh	H3K27ac_Enh
Blood & T-cell	BLD.CD4.CD25M.IL17M.PL.TPC			Digestive	GI.CLN.MUC	H3K4me1_Enh	H3K27ac_Enh
Blood & T-cell	BLD.CD4.CD25M.IL17P.PL.TPC			Digestive	GI.RECT.MUC.29	H3K4me1_Enh	H3K27ac_Enh
Blood & T-cell	BLD.CD4.CD25M.CD45RO.MPC			Digestive	GI.RECT.MUC.31	H3K4me1_Enh	H3K27ac_Enh
Blood & T-cell	BLD.CD4.MPC	H3K4me1_Enh	H3K27ac_Enh	Digestive	GI.STMC.MUC	H3K4me1_Enh	
Blood & T-cell	BLD.CD8.MPC	H3K4me1_Enh	H3K27ac_Enh	Digestive	GI.DUO.MUC	H3K4me1_Enh	
Blood & T-cell	BLD.CD4.NPC			Digestive	GI.ESO	H3K4me1_Enh	H3K27ac_Enh
Blood & T-cell	BLD.CD8.NPC			Digestive	GI.STMC.GAST	H3K4me1_Enh	H3K27ac_Enh
HSC & B-cell	BLD.CD14.PC			Other	PLCNT.AMN	H3K4me1_Enh	H3K27ac_Enh
HSC & B-cell	BLD.CD19.CPC			Other	KID.FET	H3K4me1_Enh	
HSC & B-cell	BLD.CD34.PC	H3K4me1_Enh		Other	LNG.FET	H3K4me1_Enh	
HSC & B-cell	BLD.MOB.CD34.PC.M	H3K4me1_Enh		Other	OVRY	H3K4me1_Enh	H3K27ac_Enh
HSC & B-cell	BLD.MOB.CD34.PC.F	H3K4me1_Enh		Other	PANC.ISLT	H3K4me1_Enh	
HSC & B-cell	BLD.CD34.CC	H3K4me1_Enh		Other	ADRL.GLND.FET	H3K4me1_Enh	H3K27ac_Enh
HSC & B-cell	BLD.CD19.PPC			Other	PLCNT.FET	H3K4me1_Enh	H3K27ac_Enh
HSC & B-cell	BLD.CD56.PC			Other	LIV.ADLT	H3K4me1_Enh	H3K27ac_Enh
HSC & B-cell	BLD.CD15.PC	H3K4me1_Enh		Other	PANC	H3K4me1_Enh	H3K27ac_Enh
Mesench	STRM.MRW.MSC	H3K4me1_Enh	H3K27ac_Enh	Other	LNG	H3K4me1_Enh	H3K27ac_Enh
Mesench	STRM.CHON.MRW.DR.MSC	H3K4me1_Enh	H3K27ac_Enh	Other	SPLN	H3K4me1_Enh	H3K27ac_Enh
Mesench	FAT.ADIP.DR.MSC	H3K4me1_Enh		ENCODE201	LNG.A549.ETOH002.CNCF	H3K4me1_Enh	H3K27ac_Enh
Mesench	FAT.MSC.DR.ADIP	H3K4me1_Enh		ENCODE201	BLD.DND41.CNCR	H3K4me1_Enh	
Myosat	MUS.SAT	H3K4me1_Enh		ENCODE201	BLD.GM12878		
Epithelial	SKIN.PEN.FRSK.FIB.01	H3K4me1_Enh	H3K27ac_Enh	ENCODE201	CRVX.HELAS3.CNCR	H3K4me1_Enh	H3K27ac_Enh
Epithelial	SKIN.PEN.FRSK.FIB.02	H3K4me1_Enh	H3K27ac_Enh	ENCODE201	LIV.HEPG2.CNCR	H3K4me1_Enh	H3K27ac_Enh
Epithelial	SKIN.PEN.FRSK.MEL.01	H3K4me1_Enh		ENCODE201	BRST.HMEC	H3K4me1_Enh	H3K27ac_Enh
Epithelial	SKIN.PEN.FRSK.MEL.03	H3K4me1_Enh	H3K27ac_Enh	ENCODE201	MUS.HSMM	H3K4me1_Enh	H3K27ac_Enh
Epithelial	SKIN.PEN.FRSK.KER.02			ENCODE201	MUS.HSMMT	H3K4me1_Enh	H3K27ac_Enh
Epithelial	SKIN.PEN.FRSK.KER.03	H3K4me1_Enh		ENCODE201	VAS.HUVEC	H3K4me1_Enh	H3K27ac_Enh
Epithelial	BRST.HMEC.35	H3K4me1_Enh		ENCODE201	BLD.K562.CNCR	H3K4me1_Enh	
Epithelial	BRST.MYO	H3K4me1_Enh		ENCODE201	BLD.CD14.MONO	H3K4me1_Enh	
Neurosph	BRN.GANGEM.DR.NRSPHR	H3K4me1_Enh		ENCODE201	BRN.NHA	H3K4me1_Enh	H3K27ac_Enh
Neurosph	BRN.CRTX.DR.NRSPHR	H3K4me1_Enh		ENCODE201	SKIN.NHDFAD	H3K4me1_Enh	H3K27ac_Enh
Thymus	THYM	H3K4me1_Enh	H3K27ac_Enh	ENCODE201	SKIN.NHEK		
Thymus	THYM.FET	H3K4me1_Enh	H3K27ac_Enh	ENCODE201	LNG.NHLF	H3K4me1_Enh	H3K27ac_Enh
				ENCODE201	BONE.OSTEO	H3K4me1_Enh	H3K27ac_Enh

Data from HaploReg v4.1 (https://pubs.broadinstitute.org/mammals/haploreg/haploreg.php)
